# Beyond Antioxidants: How Redox Pathways Shape Cellular Signaling and Disease Outcomes

**DOI:** 10.3390/antiox14091142

**Published:** 2025-09-22

**Authors:** Abdallah Alhaj Sulaiman, Vladimir L. Katanaev

**Affiliations:** 1 Translational Oncology Research Center, Qatar Biomedical Research Institute (QBRI), Hamad Bin Khalifa University (HBKU), Qatar Foundation (QF), Doha P.O. Box 34110, Qatar; asulaiman@hbku.edu.qa; 2 Translational Research Centre in Oncohaematology, Department of Cell Physiology and Metabolism, Faculty of Medicine, University of Geneva, Rue Michel-Servet 1, CH-1211 Geneva, Switzerland

**Keywords:** redox signaling, reactive oxygen species (ROS), oxidative stress, disease mechanisms, signaling pathways

## Abstract

Cellular redox pathways are critical regulators of various biological processes, including the intricate modulation of intracellular signaling pathways. This review explores how major redox enzymes—such as catalase, superoxide dismutases, glutathione peroxidases, thioredoxins, and peroxiredoxins—interact with key cellular signaling pathways, including receptor tyrosine kinase, mTORC1/AMPK, Wnt/β-catenin, TGF-β/SMAD, NF-κB, Hedgehog, Notch, and GPCR signaling. By investigating mechanisms such as ROS-mediated activation, cysteine oxidation, spatial enzyme localization, and phosphatase regulation, we demonstrate the extensive influence of redox balance on cellular signaling dynamics. Understanding these redox-dependent interactions provides insights into pathophysiological conditions ranging from cancer to fibrosis, offering novel therapeutic opportunities.

## 1. Introduction

**Figure 1:**
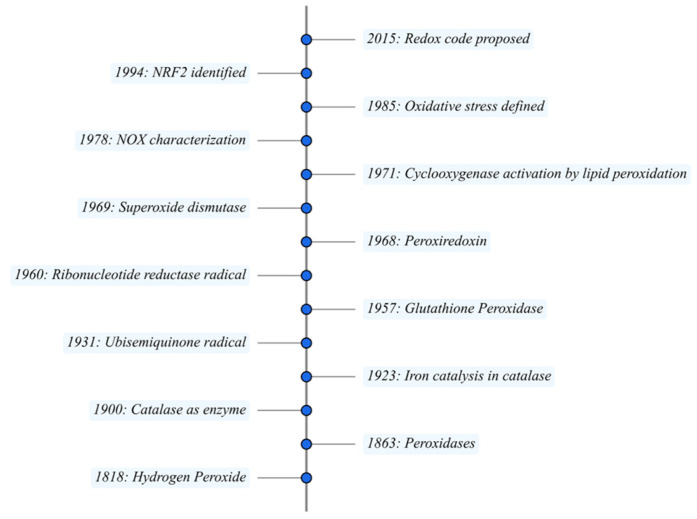
**Timeline of key redox biology discoveries:** Seminal milestones in the field of redox biology are plotted along a vertical time axis from 1818 to 2015. Figure prepared with BioRender.

Cellular redox pathways are fundamental modulators of biological processes, extending their influence far beyond simple antioxidant defense mechanisms. Accumulating evidence highlights how key redox components intricately regulate various signaling networks, profoundly impacting cellular physiology and disease states [1]. The historical milestones in the identification of critical redox molecules and enzymes (Figure 1) set the foundation for understanding their regulatory roles beyond mere detoxification.

**Figure 2:**
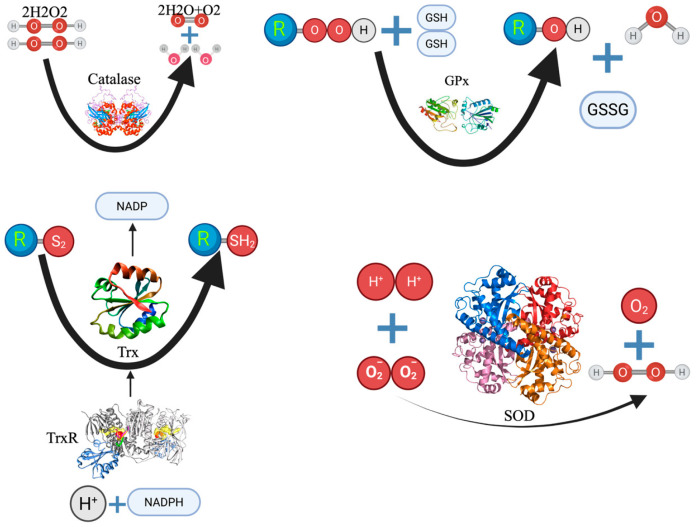
**General reaction mechanisms of several key cellular antioxidant enzymes:** Catalase decomposes H_2_O_2_ into two molecules of water (H_2_O) and one molecule of O_2_. GPx reduces H_2_O_2_ or lipid hydroperoxides (ROOH) to H_2_O or the corresponding alcohol (ROH) by oxidizing two molecules of glutathione (GSH) into glutathione disulfide (GSSG). Reduced Trx (Trx-(SH)_2_) donates electrons to oxidized protein disulfides and other free-radical targets, becoming oxidized (Trx-S_2_) in the process and maintaining redox homeostasis. TrxR transfers electrons from NADPH to oxidized Trx (Trx-S_2_), restoring it to the active dithiol form (Trx-(SH)_2_), thus sustaining Trx-dependent antioxidant and signaling functions. SOD catalyzes the dismutation of two molecules of superoxide radical (O_2_•^−^) into H_2_O_2_ and molecular O_2_. Crystal structures from PDB IDs 1ERT, 1AP5, 1GP1, 1A4E, and 3EAO. Figure prepared with BioRender.

Hydrogen peroxide (H_2_O_2_), initially synthesized by Louis Jacques Thénard in 1818, became biologically significant when Christian Friedrich Schönbein identified peroxidase activity and catalase-mediated decomposition [2,3]. Catalase (Figure 2), isolated by Oscar Loew and mechanistically clarified by Otto Warburg, provided the first insights into enzymatic redox control via iron-porphyrin centers [4,5]. In 1954, Gerschman et al. proposed that oxygen poisoning and X-irradiation act via a common mechanism [6]. Later, Glutathione peroxidases (GPx) (Figure 2) were first described by Gordon C. Mills in 1957. This discovery established the biological significance of enzymes containing selenium as essential cofactors, thereby defining their critical antioxidant functions [7,8]. Subsequent research revealed that GPx1–4 and GPx6 incorporate selenocysteine, whereas the others replace it with cysteine [9,10]. Among the selenoproteins, GPx4—originally named PIP—uniquely reduces complex phospholipid hydroperoxides to prevent lipid peroxidation and ferroptosis [11,12,13] (Table 1).

Thioredoxin (Trx) (Figure 2) was first isolated and characterized by Laurent, Moore, and Reichard in 1964, who identified it as the hydrogen donor for ribonucleotide reductase from *Escherichia coli* [14]. In the same year, Moore, Reichard, and Thelander purified the flavin-containing enzyme—coining it “Thioredoxin Reductase (Trxr)” (Figure 2)—from *E. coli* and showed that it uses NADPH (nicotinamide adenine dinucleotide phosphate) and a bound flavin adenine dinucleotide to catalyze the reduction of oxidized Trx-S_2_ (disulfide) to Trx-(SH)_2_ (dithiol form). They determined it to be a ~68 kDa flavoprotein, measured its kinetic parameters, and demonstrated its central role in driving thioredoxin-mediated reduction of disulfide substrates across various cellular processes [15]. In 1994, Griendling et al. showed that angiotensin II increases intracellular superoxide in vascular smooth muscle by stimulating NADPH- and NADH (nicotinamide adenine dinucleotide)-oxidase activity [16].

The peroxiredoxins (Prxs) were initially observed as “torin” in 1968, referring to a ~20 kDa torus-shaped protein isolated from hemoglobin-depleted human erythrocyte membranes via gentle dialysis and electron microscopy. Subsequent amino-acid sequencing and enzymatic assays revealed that torin harbors the conserved peroxidatic cysteine and functions as a thioredoxin-dependent hydroperoxide reductase, establishing its role in thiol-based redox signaling and the cellular antioxidant network [17,18,19]. Mammalian peroxiredoxins comprise six isoforms (Prx1–Prx6) grouped by mechanism into typical 2-Cys (Prx1–4), atypical 2-Cys (Prx5), and 1-Cys (Prx6), all using a conserved peroxidatic Cys and, generally, thioredoxin for regeneration [20]. Prx1/2 are mainly cytosolic (also nuclear); Prx3 is mitochondrial; Prx4 localizes to the ER/secretory pathway; Prx5 is distributed across mitochondria, peroxisomes, and the cytosol (and nucleus); and Prx6 is largely cytosolic/lysosomal and uniquely carries Ca^2+^-independent phospholipase A_2_ activity that aids membrane repair [21,22]. A knockout study indicates only partial redundancy and limited direct evidence for systematic functional complementarity of Prx6 by other family members [22].

Superoxide dismutases (SODs) (Figure 2) were first defined in 1969 by McCord and Fridovich, who showed that superoxide radicals produced the unexpected, oxygen-dependent reduction of ferricytochrome c and identified an enzyme—SOD—that catalyzes the conversion of superoxide into hydrogen peroxide and oxygen, thereby explaining the phenomenon. In that same study, they purified a copper/zinc-containing SOD from bovine erythrocytes—previously known under names such as erythrocuprein and hemocuprein—and characterized its metal cofactor and enzymatic activity [23]. Subsequent work revealed a family of SOD isoenzymes: manganese-dependent SODs in bacteria and mitochondrial matrices, iron-dependent SODs in prokaryotes and protozoa, and extracellular copper/zinc SODs in mammals, stressing the complex role of SODs in defending cells against superoxide-mediated oxidative stress [24].

The essential role of selenium in redox biology was not established until 1973, when Rotruck et al. demonstrated that GPx activity depends on selenium and that selenium is incorporated into the enzyme [7]. Forstrom, Zakowski & Tappel then identified selenocysteine as the catalytic residue in GPx, confirming it as a true selenoenzyme [25]. Subsequent work extended the family of selenium-dependent redox catalysts to include phospholipid hydroperoxide glutathione peroxidase [9] and mammalian thioredoxin reductase [26], cementing selenium’s function as a key antioxidant cofactor. In 1978, Segal and Jones characterized a novel phagocyte flavocytochrome b_558_—later recognized as the catalytic core of the NADPH oxidase complex—revealing a dedicated flavocytochrome-based system essential for microbicidal superoxide production [27,28]. Also, in 1997, Itoh et al. provided the first direct evidence that Nuclear factor erythroid 2–related factor 2 (Nrf2), when heterodimerized with small Maf proteins, binds to antioxidant response elements to drive the coordinated transcriptional induction of phase 2 detoxifying enzyme genes in response to oxidative stress [29]. In *E. coli*, Oxidation–Isotope Coded Affinity Tag-based thiol-trapping quantified oxidative modification of cysteines in hundreds of proteins, with Leichert et al. identifying a core set of targets exhibiting 30–90% oxidation across key metabolic, translational, and stress-response pathways [30]. Furthermore, in marine diatoms, Rosenwasser et al. applied quantitative mass-spectrometry to measure oxidation of 3845 cysteines, revealing approximately 300 redox-sensitive proteins involved in nitrogen assimilation and photosynthetic functions [31]. Together, these milestones and others transformed our view of redox chemistry from passive detoxification to an active regulator of metabolism, gene expression, immune function, and cell survival. Building on these discoveries, Jones and Sies present a unified “redox code” that conceptualizes how NAD/NADP redox couples and thiol/disulfide systems coordinate spatial and temporal regulation of metabolism and signaling. This code depicts kinetic thiol–disulfide switches translating redox status into protein function and localized peroxide fluxes directing processes from development to circadian rhythms [32].

This review focuses on elucidating how these critical redox pathway components interact with and regulate diverse cellular signaling pathways, affecting physiological and pathological processes. By understanding these interactions, we can better grasp the integrated nature of redox biology in maintaining cellular function and influencing disease progression.

## 2. Redox Regulation of Receptor Tyrosine Kinase Signaling

Receptor tyrosine kinases (RTKs) are single-pass transmembrane growth-factor receptors that possess intrinsic kinase domains activated upon ligand binding and receptor dimerization. This event triggers autophosphorylation of specific tyrosine residues, which subsequently recruit adaptor proteins containing SH2 (Src Homology 2) and PTB (Phosphotyrosine Binding) domains. These adaptors initiate downstream signaling cascades, predominantly Ras–MAPK (Mitogen-Activated Protein Kinase) and PI3K (phosphoinositide 3-kinase)–AKT (Ak strain transforming, aka. Protein kinase B) pathways, regulating critical cellular processes such as proliferation, survival, and metabolism [33]. Several studies have demonstrated that redox enzymes play significant roles in regulating RTK signaling (Table 2) through their influence on reactive oxygen species (ROS) and redox-sensitive cysteine residues in RTKs, as will be discussed below.

### 2.1. Redox Control of RTK Signaling Pathways: Mechanisms and Implications

Reactive oxygen species, notably H_2_O_2_, serve as central mediators in redox regulation of RTK signaling. In chronic lymphocytic leukemia cells, an imbalance between SOD2, which converts harmful superoxide radicals into H_2_O_2_, and catalase, responsible for decomposing H_2_O_2_, leads to excessive H_2_O_2_ accumulation. This increase in H_2_O_2_ activates the AXL receptor tyrosine kinase independently of its growth-factor ligand, initiating survival pathways via AKT and ERK (Extracellular signal-regulated kinase) signaling [34]. A similar principle of ROS-mediated activation is illustrated by Kato et al., who demonstrated that UV (ultraviolet)-induced free radicals promote RET (REarranged during Transfection, an RTK) activation through oxidative modification of cysteine residues. Preventing this modification via mutation or antioxidant enzymes like copper/zinc-superoxide dismutase 1 (Cu/Zn-SOD1) inhibits this activation, highlighting the direct effect of redox components on the RTK [35]. ROS not only directly activate RTKs but also modulate signaling duration and strength by transiently inhibiting protein tyrosine phosphatases (PTPs), key negative regulators of RTKs. Juarez et al. showed the essential role of cytosolic SOD1 in converting superoxide radicals into H_2_O_2_, transiently inactivating PTPs such as protein tyrosine phosphatase 1B (PTP1B). Inhibition of SOD1 reduces H_2_O_2_, maintains active PTPs, and significantly attenuates growth-factor-driven RTK signaling. This emphasizes SOD1 as a critical redox modulator, balancing RTK signaling intensity via phosphatase regulation [36].

Redox enzymes also regulate RTK activity through their spatial localization under oxidative conditions. In human endothelial cells, physiological ROS drive thioredoxin-interacting protein (TXNIP) to relocalize from the nucleus to the plasma membrane and, in a TXNIP-dependent manner, recruit Trx1 to the membrane; ROS thereby promote TXNIP–Trx1 complex formation at the membrane. This complex mediates ROS-dependent transactivation of VEGFR2 (vascular endothelial growth factor receptor 2), leading to ERK1/2 activation. Functionally, TXNIP/Trx1 are required for endothelial migration to VEGF/ROS/TNF-α (tumor necrosis factor-alpha) and for survival under oxidative stress; disrupting this trafficking/interaction blunts VEGFR2 signaling and increases apoptosis [37]. Additionally, Laukkanen et al. expanded this regulatory mechanism to the extracellular space, showing that extracellular superoxide dismutase 3 (SOD3) modulates RTK signaling broadly in anaplastic thyroid cancer cells. Extracellular SOD3 expression enhances phosphorylation of multiple RTKs and downstream Src family kinases, altering the transcriptional regulation of small GTPase modulators and impacting cellular growth signaling networks. This highlights a complex extracellular dimension of redox control over RTKs [38].

Intracellular redox regulation also involves mitochondrial enzymes like MnSOD (SOD2), which indirectly modulates RTK activation. In murine fibroblasts, exogenous H_2_O_2_ activates the PDGFRβ (platelet-derived growth factor receptor-β) and Src kinase, triggering the apoptotic Ras–MAPK cascade. Elevated mitochondrial MnSOD activity counters this by reducing superoxide and subsequent H_2_O_2_ levels, preventing excessive PDGFRβ and Src phosphorylation, blunting ERK signaling, and substantially mitigating apoptosis. Thus, mitochondrial ROS handling constitutes another crucial aspect of redox regulation, safeguarding cells against oxidative stress-induced RTK overactivation [39]. Collectively, these findings illustrate how intricate redox regulation through ROS generation, cysteine oxidation, spatial enzyme localization, and modulation of phosphatase activity orchestrates precise control over RTK signaling, profoundly influencing cell physiology and survival (Table 2).

### 2.2. Redox as a Modulator of RTK Signaling in Disease Contexts

In cancer cells, manipulation of redox pathways can strategically disrupt RTK-driven survival signals. For instance, hyperforin treatment in melanoma cells harboring BRAFV600E mutation induces heme oxygenase-1 (HMOX1) expression, increases transferrin expression, and lowers glutathione peroxidase-4 to trigger iron-dependent lipid peroxidation and autophagy. This heightened oxidative state suppresses AXL receptor tyrosine kinase expression; in the same setting, AXL reduction is observed after hyperforin exposure, although the study did not test whether AXL suppression is directly caused by oxidative stress or HMOX1 [40], thereby impairing downstream AKT/ERK signaling crucial for melanoma cell invasion and metastasis. Similarly, in non-small cell lung cancer (NSCLC), tumor cells resistant to epidermal growth factor receptor (EGFR) inhibitors (erlotinib, osimertinib) depend heavily on the NRF2-mediated antioxidant response to maintain low intracellular ROS levels via upregulation of GPx4 and SOD2. By silencing NRF2 or inhibiting GPx4, ROS accumulates, overcoming resistance and restoring sensitivity to EGFR inhibition. This highlights how cancer cells exploit redox modulation of RTK signaling as a survival mechanism, presenting potential therapeutic targets in the NRF2-GPX4/SOD2 axis [41].

Beyond cancer, in a methamphetamine mouse model, within the prefrontal cortex, novel object-evoked ERK1/2 phosphorylation (that is, the increase in ERK1/2 phosphorylation triggered by exposure to a new object during the Novel Object Recognition Test) is suppressed. GPx1 knockout significantly enhances this methamphetamine-induced reduction at 1 and 28 days after withdrawal from repeated methamphetamine administration, whereas GPx1 overexpression prevents the reduction and preserves ERK1/2 activation, in parallel with rescue of recognition memory [42]. Additionally, peroxiredoxins—specifically Prx2—in balloon-injured rat carotid arteries and human atherosclerotic lesions modulate RTK pathways to coordinate vascular repair. In vascular smooth muscle cells, Prx2 oxidation causes hydrogen peroxide accumulation that inhibits PDGFRβ-targeting protein tyrosine phosphatases, resulting in persistent PDGFRβ autophosphorylation and promoting detrimental proliferation. In endothelial cells, Prx2 loss leads to oxidative inactivation of VEGFR2, disrupting essential vascular repair, rescued by a peroxiredoxin-mimetic [43]. However, enhancing antioxidant enzyme activity does not universally alter RTK-derived outcomes, as evidenced by clinical findings. In pregnant women at high risk for preeclampsia, selenium supplementation successfully elevated plasma GPx activity yet failed to influence circulating sFlt-1 (soluble fms-like tyrosine kinase-1, soluble VEGFR-1), an RTK-derived anti-angiogenic factor. This indicates the complexity and context-dependence of redox modulation of RTKs, suggesting that antioxidant interventions must be tailored specifically to disease settings and biological systems [44]. Understanding the specificity of these interactions provides critical insights for developing targeted therapeutic strategies across diverse disease states.

### 2.3. Redox Regulation of RTK Signaling via PTPs

PTPs serve as crucial modulators of RTK signaling, and their activity can be intricately controlled by cellular redox states. Central redox enzymes, including GPx1, SOD2, and thioredoxin reductase 1, have emerged as key regulators in this context, modulating PTP activity through distinct mechanisms.

GPx1 acts as an essential protective enzyme against oxidative stress, as shown in a mouse model of cigarette smoke-induced lung inflammation. Smoke exposure reduces GPx1 levels, triggering increased protein tyrosine phosphorylation and inflammatory responses. GPx1 directly interacts with and activates PTP1B, which subsequently activates protein phosphatase 2A (PP2A). The activated PP2A then suppresses multiple RTK-driven inflammatory pathways. Restoration of GPx1 activity, either genetically or pharmacologically, rebalances these phosphorylation events, reduces inflammatory cell infiltration, and preserves lung architecture, highlighting a GPx1–PTP1B–PP2A signaling axis crucial for controlling the kinase-mediated inflammatory signaling [45,46,47,48]. Complementing this, mitochondrial antioxidant enzyme SOD2 influences RTK signaling through a parallel yet distinct PTP-dependent mechanism. In fibroblast-specific SOD2 knockout mice, loss of SOD2 increases mitochondrial superoxide levels, activating both PTP1B and phosphatase and tensin homolog (PTEN). This dual phosphatase activation results in dephosphorylation of insulin-like growth factor 1 receptor (IGF-1R) and phosphatidylinositol-3,4,5-triphosphate (PIP_3_), significantly reducing downstream AKT phosphorylation and impairing fibroblast proliferation. Consequently, this redox shift leads to decreased collagen synthesis and age-related skin thinning. Importantly, pharmacological inhibition of these phosphatases rescues these effects, directly linking mitochondrial ROS elevation to altered RTK signaling via PTPs and highlighting potential therapeutic targets against oxidative stress-mediated aging processes [49].

Further insight into the redox-PTP-RTK interplay is provided by studies involving the Trx antioxidant system. The cytosolic Trx system, specifically comprising TrxR1, Trx1, and thioredoxin-related protein 14 (TRP14), reactivates oxidized PTP1B. Loss or inhibition of TrxR1 leads to persistent oxidation (inactivation) of PTP1B, usually at the active-site cysteine within the HC(X)_5_R motif, allowing prolonged phosphorylation (activation) of PDGFRβ, thereby enhancing cellular proliferation responses to PDGF-BB (Platelet-Derived Growth Factor-BB). Restoration of Trx system components re-establishes PTP1B activity, normalizes PDGFRβ phosphorylation, and restores regulated RTK signaling. Thus, TrxR1 modulates RTK signaling termination through redox-sensitive reactivation of PTP1B [50].

On top of that, electrophilic modifications represent another layer of complexity, controlling RTK signaling via direct redox-dependent inhibition of PTPs and TrxR1. Endogenous reactive electrophiles—including lipid peroxidation products like 4-hydroxynonenal (4-HNE), methylglyoxal, and especially the oxidant peroxymonocarbonate (a potent oxidant formed from hydrogen peroxide and bicarbonate)—target critical cysteine and selenocysteine residues, transiently inhibiting PTP activity. This inhibition leads to enhanced RTK phosphorylation and sustained downstream signaling [51]. Concurrently, electrophile-modified KEAP1 (Kelch-like ECH-associated protein 1) activates the NRF2 antioxidant response, which boosts expression of antioxidant enzymes, including Trx, GPx systems, and SODs. This antioxidant induction serves as a feedback mechanism, ultimately restoring PTP activity to terminate RTK signaling. These electrophile-dependent redox events, however, strongly depend on electrophile concentration, duration of exposure, and subcellular context, emphasizing the complexity and fine-tuning inherent to redox-based regulation of RTK and PTP activity [51]. Together, these studies illustrate a sophisticated and highly regulated network in which distinct redox enzymes interact with phosphatases to dynamically modulate RTK-driven pathways across diverse biological contexts.

### 2.4. Thioredoxin Regulation of RTK Signaling: From Receptor Biogenesis to Downstream Activation

Thioredoxins play important roles in RTK signaling, affecting pathways at multiple regulatory points, including receptor maturation and downstream signaling cascades. Sartelet et al. initially revealed that cytosolic Trx1 positively influences AKT signaling in neuroblastoma. Assessing primary neuroblastoma samples and cell lines, the authors found that Trx1 expression positively correlated with the activation of phosphorylated AKT as well as upstream RTKs (e.g., IGF1 receptor, tropomyosin receptor kinase B, VEGFR1) and downstream targets such as phosphorylated p70S6K (70 kDa ribosomal S6 kinase). Functional assays demonstrated that recombinant Trx1 increases AKT phosphorylation, promotes cell cycle progression (G2/M transition), and enhances cell survival under conditions of therapeutic stress. These findings established Trx1 as an important regulator of RTK-driven AKT signaling and stressed its potential as a therapeutic target [52]. Expanding the role of thioredoxins beyond cytosolic modulation, Dong et al. later provided evidence of a distinct ER-localized regulatory mechanism involving anterior gradient 2 (AGR2), an ER-resident thioredoxin-like protein. AGR2 was shown to be critical for the proper maturation and trafficking of EGFR from the ER to the plasma membrane [53]. AGR2 forms temporary mixed-disulfide intermediates with nascent EGFR molecules, forming mixed-disulfide intermediates consistent with facilitating disulfide-dependent folding and subsequent surface delivery. Loss of AGR2 or mutation of its thioredoxin-active site cysteine severely impaired EGFR maturation, drastically reducing EGFR surface expression, receptor phosphorylation, and downstream activation of genes like EGR1 (early growth response 1) and c-Fos. Restoration experiments confirmed that the redox-active thioredoxin domain of AGR2 is essential for EGFR functionality [53].

Collectively, these studies indicate the complex interplay between redox enzymes and RTK signaling, demonstrating how redox status intricately governs receptor activation, downstream signaling cascades, phosphatase regulation, and receptor biogenesis. This dynamic control profoundly influences key biological outcomes across diverse physiological and pathological contexts. Deeper understanding of these redox-regulated networks provides promising opportunities for targeted therapeutic interventions in diseases driven by dysregulated RTK signaling.

**Table 2:** **Summary of Redox Enzyme Systems Modulating RTK Signaling:** In this table, “↑” denotes an increase and “↓” a decrease in enzyme activity or metabolite level, “→” indicates a causal sequence (‘leads to’ or ‘results in’) in the mechanism column, while arrows in the mechanism column indicate the sequence of molecular events leading to RTK modulation. It shows how shifts in redox enzymes—SOD1–3, GPx, Prx2, the thioredoxin system, and AGR2—alter ROS levels or cysteine redox states to promote or inhibit RTKs such as AXL, RET, EGFR, and PDGFRβ. The summary covers the contexts in which these changes were observed (from CLL (chronic lymphocytic leukemia) and NSCLC cell lines to vascular injury and fibroblast models) and their consequences for cell survival, proliferation, drug resistance, inflammation, and tissue repair in disease-relevant settings.

Redox Enzyme/System	RTK Target (Direct or Indirect)	Mechanism of Regulation	Biological/Cellular Context	Disease/Physiological Relevance	Ref.
SOD2↑/Catalase ↓	AXL	SOD2 ↑ + Catalase↓ → H_2_O_2_ ↑ → AXL oxidation/phosphorylation → AKT/ERK → survival ↑	CLL cells exhibiting SOD2–catalase imbalance	Promotes CLL cell survival and proliferation via AXL-mediated prosurvival pathways	[34]
UV-induced ROS ↑/Cu/Zn-SOD1 ↑	RET	UV → ROS↑ → RET Cys-ox → autophosphorylation → activation; SOD1 ↑ or Cys → Ser → no activation	Cultured cells exposed to UV light	Demonstrates ROS-driven RET activation mechanism with relevance to UV-induced carcinogenesis	[35]
Cytosolic SOD1 ↑	Growth-factor RTKs (e.g., EGFR)	SOD1 ↑ → O_2_•^−^ → H_2_O_2_ ↑ → PTP1B-ox (inactive) → sustained RTK phosphorylation; SOD1 ↓ → H_2_O_2_ ↓ → PTP1B active → RTK signaling↓	Growth-factor-stimulated cells	Controls signaling duration/intensity; relevant to tumor cell proliferation	[36]
Extracellular SOD3 ↑	Multiple RTKs	SOD3 ↑ → extracellular O_2_^−^ → altered redox milieu → ↑phosphorylation of RTKs → Src kinase activation → transcriptional shifts in small GTPase modulators	Anaplastic thyroid cancer cells	Modulates growth-signaling networks in aggressive thyroid cancer, with potential impact on tumor progression and therapy response	[38]
Mitochondrial SOD2 ↑	PDGFβ receptor, Src kinase	MnSOD ↑ → H_2_O_2_ ↓ → ↓ PDGFRβ & Src phosphorylation → ↓ ERK → reduced apoptosis; Exogenous H_2_O_2_ → PDGFRβ/Src activation → Ras–Raf–MEK–ERK → apoptosis	Murine fibroblasts treated with exogenous H_2_O_2_	Protects cells from oxidative stress–induced RTK overactivation and apoptosis; relevant to tissue injury and fibrotic disease models	[39]
Hyperforin-induced HMOX1 ↑ → lipid peroxidation & autophagy ↑	AXL	Hyperforin → HMOX1 ↑ → ↑ lipid peroxidation & autophagy → AXL expression ↓ → AKT/ERK signaling ↓	BRAF-mutant melanoma cells treated with hyperforin	Impairs melanoma cell invasion and metastasis by suppressing AXL-driven survival pathways	[40]
NRF2 ↑ (GPx4 ↑, SOD2 ↑) → NRF2 silencing/GPx4 inhibition	EGFR	NRF2 ↑ → GPx4 & SOD2 ↑ → ROS ↓ → EGFR inhibitor resistance; GPx4 inhibition → ROS ↑ → restores sensitivity to EGFR inhibitors	Erlotinib/osimertinib-resistant NSCLC cell lines	Overcoming acquired resistance to EGFR-targeted therapies in NSCLC	[41]
Prx2 oxidation ↓/restoration ↑	PDGFRβ; VEGFR2	Prx2 oxidation → impaired VEGFR2 signaling (EC repair); Prx2 restoration → normalization of VEGFR2 signaling	Vascular smooth muscle cells and endothelial cells in balloon-injured rat carotid arteries and human atherosclerotic lesions	Drives pathological neointimal growth and defective repair in vascular injury/atherosclerosis; restoration of Prx2 activity inhibits hyperplasia and promotes healing	[43]
Selenium supplementation → ↑ plasma GPx activity	sFlt-1 (soluble VEGFR-1)	GPx activity ↑ → oxidative stress ↓; no measurable change in circulating sFlt-1 levels	Pregnant women at high risk for preeclampsia	Shows that boosting antioxidant enzymes does not always alter RTK-derived factors in vivo, underscoring context dependence of redox interventions in preeclampsia	[44]
GPx1 ↓/restoration ↑	Multiple RTKs	smoke → GPx1 ↓ → ↑ Tyr phosphorylation → inflammation; GPx1 restoration → GPx1 binds → PTP1B↑→ ↓ RTK phosphorylation → ↓ inflammation	Mouse model of cigarette smoke–induced lung inflammation	Demonstrates GPx1–PTP1B–PP2A axis in suppressing RTK-driven inflammatory signaling and preserving lung structure	[45]
Mitochondrial SOD2 ↓	IGF-1R	SOD2 ↓→ O_2_^−^ ↑ → PTP1B → IGF-1R & PIP_3_ dephosphorylation → ↓ AKT phosphorylation → ↓ fibroblast proliferation	Fibroblasts from SOD2 knockout mice	Decreased collagen synthesis and age-related skin thinning due to impaired proliferation	[49]
Cytosolic Trx system (TrxR1/Trx1/TRP14) ↑	PDGFRβ	TrxR1/Trx1/TRP14 ↑ → reduction PTP1B → PDGFRβ dephos phorylation;TrxR1 inhibition → PTP1B remains inactive → PDGFRβ phosphorylation → ↑ proliferation	PDGF-BB-stimulated cells (e.g., fibroblasts)	Ensures timely termination of PDGFRβ signaling; dysregulation may drive hyperproliferative diseases (e.g., fibrosis, cancer)	[50]
ER-resident Trx-like AGR2 ↑/loss	EGFR	AGR2 ↑ → mixed-disulfide with nascent EGFR → plasma-membrane trafficking → EGFR expression → EGR activation; AGR2 loss or Cys-active-site mutation → EGFR misfolding → ↓ surface EGFR & signaling	Cultured EGFR-expressing mammalian cells; ER maturation stage	Critical for EGFR-driven proliferation and gene expression in cancers; AGR2 dysfunction impairs EGFR signaling and may affect tumor growth	[53]

## 3. mTORC1/AMPK Metabolic Sensing Axis

mTORC1 (mechanistic Target of Rapamycin Complex 1) is a conserved serine–threonine kinase complex—composed of mTOR (mechanistic target of rapamycin), Raptor (Regulatory-associated protein of mTOR), mLST8 (mammalian lethal with SEC13 protein 8), PRAS40 (proline-rich Akt substrate of 40kDa), and Deptor (DEP domain–containing mTOR-interacting protein)—that integrates inputs from growth factors, amino acids, energy status, and stress to balance anabolic and catabolic processes. Upon activation, mTORC1 phosphorylates downstream effectors such as 4E-BP1 (eIF4E(eukaryotic translation initiation factor 4E)-binding protein 1) and S6K1, thereby promoting protein synthesis and ribosome biogenesis, while simultaneously suppressing autophagy through inhibition of ULK1 (Unc-51-like kinase 1) [54]. Its upstream regulation involves signals from the PI3K–AKT and Ras–ERK pathways converging at the tuberous sclerosis complex (TSC1/2)–Rheb (Ras homolog enriched in brain) axis, nutrient availability via Rag GTPases, and energy levels via AMPK (AMP-activated protein kinase) signaling [54]. Multiple studies have increasingly highlighted that cellular redox state profoundly influences mTORC1 activity.

### 3.1. Organelle Redox Checkpoints: Peroxisomal and Lysosomal Regulation of mTORC1

**Figure 3:**
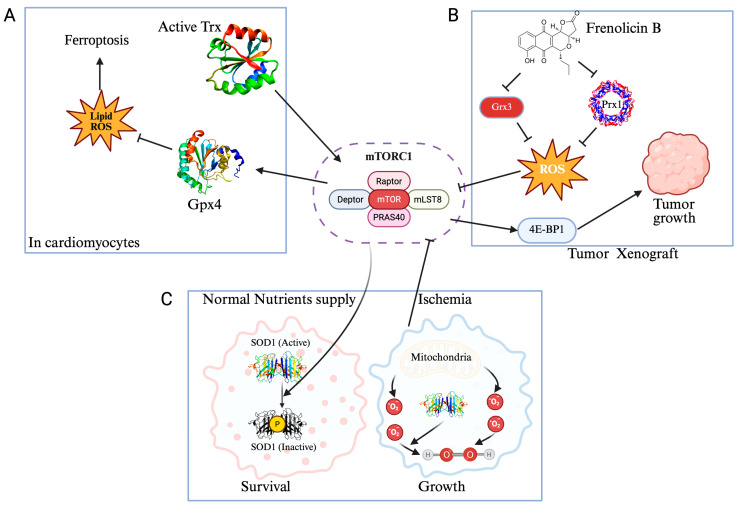
**Redox regulation of mTORC1 in distinct cell contexts:** (**A**) In cardiomyocytes, exosomes from hypoxia-preconditioned MSCs deliver active Trx, which activates mTORC1 and induces GPx4 to limit lipid peroxidation and iron accumulation, thereby preventing doxorubicin-triggered ferroptosis. (**B**) In cancer xenografts, frenolicin B inhibits Prx1 and Grx3 to raise ROS, leading to suppression of mTORC1 and impairment of cancer cell proliferation. (**C**) Under nutrient-replete vs. nutrient-depleted conditions, active mTORC1 docks on SOD1 and phosphorylates it at Thr40 to transiently blunt O_2_•^−^ dismutation and permit low-level ROS-driven proliferation, whereas nutrient withdrawal triggers SOD1 dephosphorylation and reactivation to bolster antioxidant defenses; under ischemia, mTORC1 turns off, so SOD1 loses its phosphate and becomes fully active. Active SOD1 then clears excess superoxide, protecting cells from oxidative damage and helping them survive. Figure prepared with BioRender.

Cellular organelles, notably lysosomes and peroxisomes, have emerged as critical nodes where redox status significantly impacts mTORC1 signaling. Walker et al. reviewed the dual roles of peroxisomes as both sources and sinks of ROS and reactive nitrogen species (RNS), facilitated by enzymes such as acyl-CoA oxidase, inducible nitric oxide synthase, catalase, peroxiredoxins, and glutathione peroxidases. They detailed how peroxisomal ROS directly activate ATM (Ataxia-telangiectasia mutated) kinase, which, when localized to peroxisomes through interaction with PEX5 (peroxisomal biogenesis factor 5, protein crucial for importing proteins into the peroxisomes [55]), phosphorylates PEX5 and promotes its ubiquitination. Crucially, activated ATM also suppresses mTORC1 signaling via the AMPK–TSC2 pathway, alleviating ULK1 inhibition and promoting the autophagic clearance of damaged peroxisomes (pexophagy). This ATM-mediated regulation involves multiple ubiquitin-dependent mechanisms engaging autophagy receptors p62 and NBR1 (neighbor of BRCA1 gene 1) [56]. Further illustrating the impact of peroxisomal ROS on mTORC1 regulation, Ye et al. identified frenolicin B, an antibiotic-derived compound, as a potent inhibitor of peroxiredoxin Prx1 and glutaredoxin Grx3. This inhibition markedly elevates intracellular ROS levels, subsequently activating the peroxisome-associated TSC complex to inhibit mTORC1 signaling, as evidenced by reduced phosphorylation of the translation regulator 4E-BP1. Such redox-driven inhibition of mTORC1 signaling impaired cancer cell proliferation in vitro and significantly reduced tumor growth in mouse xenograft models (Figure 3) [57]. Expanding on the role of peroxisomes, it was shown that the loss of the peroxisomal antioxidant enzyme catalase results in elevated intracellular H_2_O_2_, which subsequently causes lysosomal membrane permeabilization and release of cathepsins. This disruption of lysosomal integrity compromises autophagic flux and leads to pronounced cellular senescence, underscored by enhanced phosphorylation of the ribosomal protein S6, indicative of activated mTORC1 signaling. Importantly, therapeutic intervention using the antioxidant N-acetylcysteine or the mTORC1 inhibitor rapamycin effectively restored lysosomal function and normalized autophagic activity [58]. While the preceding examples highlight how peroxisome- and lysosome-derived ROS feed directly into mTORC1 regulation via ATM-AMPK-TSC2 signaling and pexophagy, emerging evidence shows that the same redox–mTORC1 circuitry operates at the level of whole tissues and disease models.

### 3.2. Redox–mTORC1 Crosstalk Across Tissues and Pathologies

In ovarian follicles, Kumar et al. revealed that GPx1—a selenoprotein that reduces H_2_O_2_ to water—is maintained by IGF-1 signaling. IGF-1 suppresses nonsense-mediated mRNA decay (NMD), ensuring proper incorporation of selenocysteine into GPx1. When mTORC1 is pharmacologically inhibited, NMD (the involvement of NMD in this setting is inferred rather than directly demonstrated) is unleashed, GPx1 levels fall, ROS accumulate in follicular-fluid cells, and oocyte maturation is impaired [59]. Polycystic kidney disease offers another lens on the redox–mTORC1 interplay. Agborbesong et al. showed that loss of peroxiredoxin 5 (Prx5)—a thiol-peroxidase that detoxifies peroxides—elevates ROS in autosomal-dominant polycystic kidney disease models. Elevated ROS activates mTORC1 (monitored by S6 phosphorylation and ERK signaling), driving cyst-lining epithelial proliferation [60]. Also, in the heart, Yu et al. discovered that exosomes from hypoxia-preconditioned mesenchymal stem cells (MSCs) carry high levels of Trx1. When transferred to cardiomyocytes, Trx1 activates mTORC1 (evidenced by phosphorylation of S6K, S6, and 4E-BP1), driving expression of GPx4. Then, GPx4 limits lipid peroxidation and iron accumulation, thereby protecting against doxorubicin-induced ferroptosis both in vitro and in vivo. Knockdown of Trx1 or treatment with rapamycin inhibits mTORC1 activation and the anti-ferroptotic effect (Figure 3) [61].

Genomic analyses in hepatocellular carcinoma (HCC) further tie redox proteins to mTORC1: Cho et al. used The Cancer Genome Atlas (TCGA) to show that tumors overexpressing Trx, TrxR1, and related family members exhibit poorer overall survival. Gene set enrichment analysis of these high-expression tumors revealed pronounced upregulation of mTORC1 hallmark genes [62]. Finally, Wong and Hagen examined how prolonged hypoxia modulates the thioredoxin inhibitor TXNIP. Under low oxygen, TXNIP is initially downregulated but then rebounds to above-baseline levels independent of hypoxia-inducible factors (HIFs). They pinpointed mTORC1’s control of the 4E-BP1/eIF4E translation axis as the key switch: active-site mTOR inhibition with the pp242 compound, which blocks 4E-BP1 phosphorylation, upregulates TXNIP, whereas rapamycin (primarily as an S6K inhibitor) does not; overexpression of eIF4E prevents the hypoxic TXNIP rebound. This work connects mTORC1-mediated translational control to thioredoxin inhibition under metabolic stress [63]. Together, these studies weave a unifying narrative: redox-sensitive enzymes and mTORC1 engage in reciprocal regulation across physiological and pathological contexts, offering multiple nodes for therapeutic intervention.

### 3.3. mTORC1 as a Redox Rheostat: From Proliferation to Stress Resistance

Cells must constantly balance growth signals against oxidative stress. Under nutrient-rich conditions, active mTORC1 docks onto and phosphorylates SOD1 at Thr40 (Ser39 in yeast), transiently blunting its conversion of superoxide (O_2_^−^) into H_2_O_2_. This deliberate dampening allows low-level ROS to act as secondary messengers that drive proliferation. When nutrients dwindle—or when rapamycin blocks mTORC1—SOD1 is promptly dephosphorylated and reactivated, bolstering antioxidant defenses to detoxify excess ROS. This elegant “on/off” switch contributes to cancer cells’ ability to toggle between rapid growth and survival in poorly perfused, ischemic niches (Figure 3) [64]. Kong & Chandel underline the evolutionary breadth of this mTORC1–SOD1 module: by relieving SOD1 inhibition under hypoxia or starvation, cells stave off lethal oxidative damage while preserving mitochondrial ROS signals needed for adaptation. They suggest that clinical “rapalogs” may sometimes backfire—paradoxically arming tumors with stronger antioxidant defenses—and they spark the question of whether mTORC1 similarly inactivates other antioxidant enzymes (Figure 3) [65].

Interestingly, neurons repurpose the same switch in the “NiMA” (nutrient-inhibition of mitochondrial activity) pathway. Lysosomal mTORC1 phosphorylates SOD1 to couple insulin/amino-acid cues with mitochondrial DNA (mtDNA) replication and oxidative phosphorylation. Loss of tau (key factor in Alzheimer’s disease pathogenesis)-mediated tuberous sclerosis complex recruitment hyperactivates SOD1, driving mtDNA over-replication and excessive respiration. Inhibiting SOD1 with compound ATN-224 restrains mtDNA synthesis and lowers brain O_2_ consumption in mice, while human Alzheimer’s samples show elevated SOD1 activity—pointing to a maladaptive redox–mTORC1 axis in neurodegeneration [66]. Finally, in glioblastoma under dual nutrient/oxygen stress, mTORC1 inhibition (via rapamycin) unleashes SOD1’s detoxifying power, safeguarding tumor cells from ROS-induced death; by contrast, TSC2 knockdown activates mTORC1 and reduces SOD1 activity under starvation. Conversely, knocking out or pharmacologically blocking SOD1 spikes intracellular ROS, depletes NADPH, and re-sensitizes tumors to metabolic challenge—even blunting rapamycin’s protective effect. Together, these data argue that co-targeting mTORC1 and SOD1 could change the situation toward oxidative collapse in hard-to-treat gliomas [67].

### 3.4. SOD1 Inhibition as a Convergence Point for mTORC1 Suppression and Autophagy

Across neurodegenerative and cancer contexts, SOD1 perturbation is associated with mTORC1 inhibition and autophagic activation. In SOD1-linked amyotrophic lateral sclerosis mouse models, Bandyopadhyaya et al. reported that mutant SOD1 motor neurons exhibit proteostatic stress (misfolding/inclusions). This stress inhibits mTORC1 activity—evidenced by reduced phosphorylation of the autophagy-suppressing kinase ULK1 at serine-757 (Ser757)—leading to hyperactive autophagic flux. Markers of this heightened autophagy include decreased levels of LC3 (microtubule-associated protein 1 light chain 3) and p62/SQSTM1 (sequestosome-1), alongside a lack of lipofuscin accumulation [68]. Notably, a parallel mechanism operates in cancer cells treated with the natural compound oleanolic acid. Liu et al. showed that oleanolic acid inactivates SOD1, causing a burst of intracellular ROS. Elevated ROS activates AMP-activated protein kinase (AMPK), a key energy sensor that directly phosphorylates and inhibits mTORC1. Inhibited mTORC1 then unleashes macroautophagy—marked by lysosomal degradation of critical metabolic enzymes—which underlies oleanolic acid’s cytotoxicity. Blocking either AMPK activation or macroautophagy stabilizes these enzymes and mitigates growth arrest [69]. In summary, mTORC1 integrates nutrient, energy, and redox cues through organelle-localized sensors and antioxidant enzymes to finely balance growth and stress responses. Therapeutically, targeting these redox-sensitive nodes offers promising avenues to rewire mTORC1 signaling in aging, neurodegeneration, cancer, and metabolic disease.

## 4. Redox-Mediated Regulation of Wnt/β-Catenin Signaling

The canonical Wnt (Wingless/Int)/β-catenin pathway controls cell fate by regulating β-catenin stability: in the absence of Wnt, a destruction complex (Axin, APC (adenomatous polyposis coli), casein kinase 1 alpha, GSK-3β (glycogen synthase kinase-3 beta)) tags β-catenin for degradation; upon Wnt ligand binding, this complex is disassembled, allowing β-catenin to enter the nucleus and activate TCF/LEF-driven transcription [70]. Overlaying this core machinery, redox cues—from GPxs to SODs and thioredoxin proteins—act as fine-tuning switches that either buffer or amplify Wnt output in response to ROS. The emerging functions of the redox pathway components in regulating Wnt signaling may identify novel targets for drug discovery dedicated to this oncogenic signaling (Table 3), which, despite the high demand, as of today lacks any approved therapies [71,72,73].

### 4.1. Context-Dependent Roles of Glutathione Peroxidases in Wnt Signaling

In one set of experiments, Kim et al. used Nkx3.1 (NK3 homeobox 1) **^−^**/**^−^** mice that spontaneously develop prostatic intraepithelial neoplasia (PIN) to assess the role of GPx3 in the development of this precancerous state of prostate. The authors found that loss of GPx3 led to a ROS buildup and reduced SOD activity, accompanied by the increased hyperplasia of PIN. At the same time, no change in β-catenin levels or in Wnt target gene expression was observed, suggesting that raising ROS is not sufficient to activate the Wnt signaling and force progression to carcinoma [74]. By contrast, in the TRAMP model of prostate cancer (Transgenic Adenocarcinoma of Mouse Prostate), Chang et al. showed that genetic ablation of GPx3 increased prostate tumorigenesis. Although no direct assessment of the Wnt pathway levels was performed, the authors observed an accompanying increase in the levels of β-catenin and its downstream targets c-Myc, Cyclin D1, FoxA2 (Forkhead box A2), and MMP7 (matrix metalloproteinase 7), along with a decrease in Axin2, a Wnt pathway inhibitor, potentially suggesting a possible effect of GPx3 on the cancer progression-promoting Wnt/β-catenin signaling pathway [75].

Going beyond ROS scavenging, Rong et al. uncovered a peroxidase-independent regulation in zebrafish embryos: the selenoprotein GPx4 physically binds T-cell factor/Lymphoid enhancer factor (TCF/LEF) transcription factors at the Wnt target promoters, blocking their access even when GPx4’s active-site selenocysteine is mutated to cysteine. Loss of maternal GPx4 boosts nuclear β-catenin and misregulates organizer genes, altering body patterning, while GPx4 overexpression tames a Wnt reporter activity. This demonstrates that GPx enzymes can modulate Wnt not only by controlling ROS but also by directly gating the β-catenin transcriptional machinery [76]. In sum, the GPx3 studies illustrate how ROS removal can be neutral or promotive towards Wnt pathway activation, while GPx4 reveals a redox-independent layer of Wnt control through direct transcription factor sequestration. Having seen how glutathione peroxidases exert context-specific control over Wnt/β-catenin, we next examine how superoxide dismutases and thioredoxin family proteins serve as additional redox checkpoints that directly gate β-catenin signaling under metabolic and oxidative stress.

### 4.2. Superoxide Dismutases and Thioredoxin Proteins as Redox Checkpoints of Wnt/β-Catenin Signaling

High-glucose and diabetic conditions unleash a burst of superoxide—one of the primary ROS—that in turn reduces Wnt/β-catenin signaling via activation of GSK-3β.Lin et al. showed in kidney mesangial cells that high glucose activates a Ras/Rac1/ERK cascade, generating superoxide that reduces Ser9 phosphorylation on GSK-3β (lifting its inhibition), reduces the levels of Wnt5a (one of the 19 Wnt proteins), and prevents β-catenin from entering the nucleus. This sequence triggers caspase-mediated cell death. However, adding polyethylene glycol–conjugated superoxide dismutase (PEG-SOD), using the ROS inhibitor DPI (diphenyleneiodonium), or expressing a kinase-dead GSK-3β mutant or constitutively active β-catenin restores Wnt signaling and blocks apoptosis. In streptozotocin (STZ)-induced diabetic rats, systemic SOD treatment likewise lowers glomerular ROS and cell death; rescues Wnt5a expression, Ser9-GSK-3β, and nuclear β-catenin in mesangial cells; and reduces proteinuria [77]. A parallel study in diabetic mouse embryos found that maternal hyperglycemia triggers a similar superoxide surge that elevates Wnt antagonists (sFRP1 (secreted Frizzled-related protein 1), Dickkopf-1), decreases Dvl (Dishevelled) phosphorylation, boosts GSK-3β activity, and lowers both β-catenin and Wnt5a in the developing heart, leading to structural defects. Transgenic overexpression of the superoxide scavenger SOD1 abolishes ROS, normalizes Dvl/GSK-3β/β-catenin signaling, and prevents these malformations in vivo. Similarly, treatment of embryonic hearts cultured ex vivo with the SOD mimetic Tempol restores Dvl/GSK-3β/β-catenin signaling, although its ability to prevent structural defects in vivo was not tested [78].

In cancer cells, mitochondrial superoxide dismutase (MnSOD, also known as SOD2) similarly guards Wnt signaling. Jung et al. treated HCT-116 (human colorectal carcinoma cell line) with 2,3,5,6-tetramethylpyrazine, which dose-dependently lowered the levels of MnSOD. This treatment reduced levels of Wnt3a and β-catenin and caused a shift in GSK-3β phosphorylation (with less inhibitory Ser9 and more activating Tyr216), resulting in increased β-catenin degradation and reduced nuclear β-catenin [79].

The Trx family provides another redox checkpoint on Wnt/β-catenin signaling. Wu et al. showed that Trx1 is essential for bone-forming (osteogenic) differentiation of periodontal ligament stem cells under both inflammatory and diabetic stress. Knocking down Trx1 with siRNA (small interfering RNA) decreased Ser9-GSK-3β phosphorylation, lowered β-catenin levels, and impaired markers of bone formation (alkaline phosphatase, collagen-1, and osteopontin). Adding back recombinant human Trx1 restored the ROS balance, reactivated Ser9-GSK-3β/β-catenin signaling, and rescued bone repair in mouse models of periodontitis with or without diabetes [80]. Previously, Funato et al. found that a Trx-related nucleoredoxin (NRX) binds Dvl’s PDZ domain under reducing conditions and that H_2_O_2_-mediated oxidation releases Dvl to activate β-catenin–TCF/LEF signaling. In Xenopus, Dvl mRNA injection into the ventral marginal zone induces secondary axes, the effect abolished by co-injection of NRX mRNA; conversely, morpholino knock-down of NRX causes head (eye) defects, rescuable by GSK3β or dominant-negative TCF. In NIH3T3 fibroblasts, NRX RNA interference boosts the TCF/LEF reporter activity, up-regulates c-Myc/Cyclin D1, enhances BrdU (5-bromo-2′-deoxyuridine) incorporation, and promotes oncogenic transformation (especially in synergism with MEK/Ras), confirming NRX as an endogenous brake on Wnt/β-catenin-driven proliferation [81]. An effect of TrxR1 on expression of Wnt target genes in CT26 colon carcinoma cells has also been observed [82]. Collectively, these studies reflect that superoxide dismutases protect β-catenin from ROS-driven degradation in both diabetic and cancer settings, while thioredoxin family proteins—Trx1, NRX, TrxR1—may serve as molecular switches that gate Dvl or β-catenin stability. We next turn to peroxiredoxins to explore their versatile roles in modulating this pathway.

### 4.3. Peroxiredoxin Family Proteins as Modulators of Wnt/β-Catenin Signaling

Peroxiredoxins use a reactive cysteine to reduce H_2_O_2_ and thereby exert both enzymatic and non-enzymatic control over Wnt/β-catenin activity across diverse tissues and disease models. In osteoarthritic cartilage, Ma et al. [83] knocked down Prx5 in chondrocytes and found that intracellular ROS doubled. This extra ROS inhibited GSK-3β by promoting Ser9 phosphorylation, which prevented GSK-3β from tagging β-catenin for destruction. As a result, β-catenin accumulated in the nucleus—confirmed by increased activity of the TOPflash (TCF/LEF luciferase Wnt reporter assay) Wnt reporter—and turned on genes like cyclin D1 and MMP13 that drive chondrocyte proliferation and matrix breakdown. Loss of Prx5 also boosted expression of Wnt4 and its receptor Frizzled-2, creating a feed-forward loop that amplifies Wnt signaling in osteoarthritis. By contrast, Lu et al. [84] showed in colorectal cancer (CRC) cells that silencing Prx2 also raised ROS, but here the extra oxidants activated GSK-3β rather than inhibited it. Increased GSK-3β activity led to more phosphorylation of β-catenin at Ser33/37—marks for its degradation—so nuclear β-catenin levels fell. This suppressed classic Wnt target genes, including c-Myc and Survivin, and slowed tumor growth when Prx2-deficient CRC cells were grown as mouse xenografts. Taken together, the two studies show that the same ROS increase can flip the GSK-3β–β-catenin switch in opposite directions, potentially due to the involvement of different Prx isoforms (Prx5 vs. Prx2), as well as distinct cell types [83,84]. Kang et al. revealed a more direct, non-enzymatic mechanism in APC-mutant CRC. They found that Prx2 binds to the enzyme Tankyrase 1 (TNKS1) and shields its zinc ion from H_2_O_2_ damage, allowing TNKS1 to modify (PARsylate) the scaffold protein Axin1. PARsylated Axin1 is degraded by the proteasome, dismantling the β-catenin destruction complex and letting β-catenin accumulate. In mouse adenoma models, loss of Prx2 inactivated TNKS1, stabilized Axin1, and caused dramatic tumor shrinkage [85]. Thus, this work defines a thiol-peroxidase-mediated “redox switch” on the destruction complex itself.

Also, peroxiredoxins support tumor aggressiveness and epithelial–mesenchymal transition (EMT). Zheng et al. [86] found that high Prx1 levels in ovarian cancer correlated with active Wnt signaling, as judged by the gene-set enrichment analysis of TCGA. Knocking down Prx1 in Caov-3 cells reduced total β-catenin, increased the cell-adhesion protein E-cadherin, decreased the mesenchymal marker vimentin, and slowed proliferation, migration, and invasion—hallmarks of EMT suppression. In SW480 CRC cells treated with the pro-oxidant β-lapachone, Liu et al. [87] showed that overexpressing Prx5 scavenged ROS, increased inhibitory the Ser9-GSK-3β, and stabilized β-catenin, thereby protecting cells from apoptosis and tipping Bcl-2 (B-cell lymphoma 2) family proteins toward survival.

Several peroxiredoxins also sustain cancer stem-like cell (CSC) traits. Xu et al. [88] report that Prx6 is upregulated in cisplatin-resistant non-small-cell lung cancer (NSCLC) CSCs, and knocking it down lowers both CSC markers (CD133, ABCG2 (ATP-binding cassette subfamily G member 2)) and β-catenin levels. In gastric cancer, Lee et al. [89] showed that depleting Prx2—via siRNA or the inhibitor conoidin A—accelerates proteasomal degradation of β-catenin, reduces the Wnt reporter activity, and halts cell proliferation and invasion; rescuing β-catenin with a proteasome blocker or GSK-3β inhibitor restores these malignant behaviors. Chandimali et al. [90] further show that Prx2 knockdown in gefitinib-resistant NSCLC CSCs raises ROS and apoptosis, and suppresses sphere formation, migration, invasion and angiogenic signaling; miR-122–mediated PRX2 silencing similarly elevates ROS/apoptosis but more strongly shuts down Wnt, Hedgehog and Notch pathways, thereby abolishing CSC traits and tumor growth. Furthermore, Peng et al. [91] found Prx2 enriched in CD133^+^/CD44^+^ colon CSCs, where its loss increases ROS, reduces nuclear β-catenin and Wnt targets, and impairs migration, invasion, and the metastatic potential. In hepatocellular carcinoma cells, overexpression of Prx2 increased the TOPflash reporter activity, whereas knockdown of Prx2 reduced cyclin D1 and c-Myc expression, induced cellular senescence as shown by increased β-galactosidase activity, and inhibited tumor growth in xenograft models. Co-immunoprecipitation, subcellular fractionation, and immunofluorescence demonstrated that Prx2 directly binds β-catenin and facilitates its translocation into the nucleus [92]. Across these studies, peroxiredoxins emerge as versatile redox sensors and guardians of Wnt/β-catenin signaling. By tuning ROS levels, protecting key regulatory proteins, or even directly ferrying β-catenin, they ensure that Wnt activity adapts appropriately to the cellular context—promoting repair in cartilage, restraining tumor growth in some cancers, or supporting stem-like traits in others.

### 4.4. Glutathione Peroxidases as Wnt/β-Catenin-Driven Detoxification Modules

Wnt/β-catenin signaling extends beyond developmental regulation to upregulate key glutathione-dependent enzymes—GPx2 in the gut and glutathione S-transferases (GSTs) in the liver—thereby safeguarding proliferating tissues against oxidative stress. In this regard, Kipp et al. demonstrated that GPx2 is a direct transcriptional target of Wnt/β-catenin signaling: β-catenin/TCF4 overexpression increases GPx2 promoter activity by ~2–2.5-fold, and mutation of the key TCF-binding element reduces this induction by over 50%. As a selenium-dependent glutathione peroxidase, GPx2 uses glutathione to remove H_2_O_2_, acting as a barrier against oxidative stress in the intestinal epithelium. The authors propose that Wnt-driven GPx2 induction scavenges both ROS and prostaglandin E_2_-mediated β-catenin activation pathways, establishing a negative feedback loop that tempers β-catenin signaling and helps maintain controlled epithelial proliferation [93]. In the liver, Giera et al. found that activating β-catenin in mouse hepatocellular tumors (via gain-of-function Ctnnb1 mutations) dramatically boosts GSTs—specifically GSTµ2, GSTµ3, and GSTµ6—mRNAs and proteins, whereas these enzymes are down in Ras-driven tumors or livers lacking β-catenin [94]. Wnt/β-catenin emerges as a regulator of hepatic detoxification and zonation. In perivenous hepatocytes, active β-catenin maintains high phase I cytochrome P450 and phase II GST expression, not by direct promoter binding but via three modes of cross-talk with xenobiotic-sensing nuclear receptors: transcriptional induction of AhR (aryl hydrocarbon receptor), CAR (constitutive androstane receptor) and PXR (pregnane X receptor); formation of β-catenin–RXRα (retinoid X receptor alpha) complexes; and co-occupancy with these receptors at detoxification gene promoters—while RXRα expression itself remains unaltered by β-catenin status [95].

Furthermore, in a cholestatic liver-injury model using α-naphthylisothiocyanate, hepatocyte-specific deletion of β-catenin prevented the adaptive rise in total hepatic glutathione seen in wild-type mice—knockout livers remained at their lower baseline levels—and led to significant downregulation of Gstm1, Gstm2, Gstm3 and Gstm6 both before and after injury. Although Nrf2 is activated (evidenced by its nuclear accumulation and up-regulation of Nqo1 (NADPH quinone dehydrogenase 1)), this fails to restore either glutathione content or GST expression. As a result, β-catenin-deficient livers show heightened lipid peroxidation, increased hepatocyte apoptosis and necrosis, and a blunted PCNA (proliferating cell nuclear antigen)-positive regenerative response, leading to impaired tissue repair and higher mortality [96]. Together, these four studies indicate that Wnt/β-catenin signaling safeguards proliferating tissues by directly and indirectly up-regulating glutathione-dependent antioxidant enzymes—GPx2 in the gut and a suite of GSTs in the liver—and that loss of this pathway renders tissues vulnerable to the ROS-driven injury.

**Table 3:** **Summary of Redox Enzymes Modulating Wnt/β-Catenin Signaling Across Models:** This table catalogs key redox-regulating enzymes whose altered expression or activity impacts Wnt/β-catenin signaling.

Enzyme (Expression Change)	Wnt/β-Catenin Outcome	Model	Ref.
GPx3 (knockout; absent)	No change in β-catenin levels or classic Wnt target gene expression	Nkx3.1^−^/^−^ mice	[74]
GPx3 (knockout; absent)	↑ β-catenin, ↑ c-Myc, ↑ Cyclin D1, ↑ FoxA2, ↑ MMP7, ↓ Axin2	TRAMP prostate cancer model	[75]
GPx4 (maternal loss; overexpression)	Loss: ↑ nuclear β-catenin & misregulated organizer genes Overexpression: suppressed Wnt reporter activity	Zebrafish embryos	[76]
SOD (PEG-SOD treatment/systemic SOD↑)	Restores Wnt5a expression, Ser9-GSK-3β phosphorylation, and nuclear β-catenin; prevents apoptosis; reduces proteinuria	High-glucose–treated mesangial cells & STZ-diabetic rats	[77]
SOD1 (overexpression or Tempol treatment)	Normalizes Dishevelled phosphorylation; restores β-catenin and Wnt5a levels; prevents cardiac malformations	Maternal hyperglycemic mouse embryos	[78]
SOD2↓ (Inhibition by TMP)	↓ Wnt3a & total β-catenin; GSK-3β p-Ser9↓ and p-Tyr216↑ → accelerated β-catenin degradation and loss of nuclear β-catenin	HCT-116 colon cancer cells	[79]
Trx1 (knockdown↓ by siRNA; rescue↑ via rhTrx1)	Knockdown: ↓ Ser9-GSK-3β phosphorylation, ↓ β-catenin & osteogenic markers (ALP, COL1, OPN); Rescue: restores Ser9-GSK-3β/β-catenin signaling and bone repair	PDLSCs under inflammatory/diabetic stress & mouse periodontitis models	[80]
NRX (overexpression/depletion)	Overexpression inhibited ectopic secondary axis formation; knockdown induced eye-absent head defects (rescued by GSK3β/dnTCF) and elevated Wnt signaling (↑ TCF/LEF reporter activity, c-Myc, Cyclin D1, BrdU incorporation, focus formation)	Xenopus embryos; NIH3T3 fibroblasts	[81]
TrxR1 (knockdown↓) & Sep15 (knockdown↓; combined)	Combined depletion → up-regulation of Wnt/β-catenin–related genes (Prl2c2, Tnc)	CT26 colon cancer cells	[82]
PRX5 (knockdown; expression↓)	↑ ROS → GSK-3β inhibition (Ser9-p) → nuclear β-catenin accumulation; ↑ cyclin D1 & MMP13; ↑ Wnt4 & Frizzled-2 feed-forward amplification of Wnt signaling	Osteoarthritic chondrocytes	[83]
PRX2 (silenced; expression↓)	↑ ROS → GSK-3β activation → ↑ β-catenin Ser33/37 phosphorylation → ↓ nuclear β-catenin → suppressed c-Myc & Survivin; slowed tumor growth	CRC cells in mouse xenografts	[84]
PRX1 (knockdown; expression↓)	↓ Total β-catenin; ↑ E-cadherin; ↓ vimentin; slowed proliferation, migration, and invasion (EMT suppression)	Caov-3 ovarian cancer cells	[86]
PRX5 (overexpression; expression↑)	ROS scavenging → ↑ Ser9-GSK-3β phosphorylation → β-catenin stabilization → protection from apoptosis & pro-survival Bcl-2 shift	SW480 CRC cells treated with β-lapachone	[87]
PRX6 (up in CSCs; knockdown↓)	Knockdown: ↓ CD133 & ABCG2 (CSC markers) and ↓ β-catenin	Cisplatin-resistant NSCLC cancer stem cells	[88]
PRX2 (depletion; expression↓)	↑ β-catenin proteasomal degradation → ↓ Wnt reporter activity → ↓ proliferation & invasion (rescued by proteasome blocker or GSK-3β inhibitor)	Gastric cancer cells (siRNA/conoidin A)	[89]
PRX2 (knockdown↓; rescue via miR-122 (microRNA-122)↑)	PRX2 knockdown: ↑ ROS & apoptosis; ↓ proliferation, sphere-formation, migration, invasion, angiogenesis; no significant Wnt/Hedgehog/Notch inhibition. miR-122 silencing of PRX2: further ↑ ROS & apoptosis; strong suppression of Wnt/β-catenin, Hedgehog, and Notch pathways; abrogated CSC traits and in vivo tumor growth	Gefitinib-resistant NSCLC CSCs	[90]
PRX2 (knockdown; expression↓)	↑ ROS; ↓ nuclear β-catenin and Wnt target expression; impaired migration, invasion, and metastatic potential	CD133^+^/CD44^+^ colon cancer stem cells	[91]
PRX2 (overexpression ↑; knockdown ↓)	Overexpression increased TOPflash reporter activity; knockdown reduced cyclin D1 and c-Myc expression, induced senescence (↑ β-galactosidase), inhibited tumor growth; PRX2 binds β-catenin and promotes its nuclear translocation	HCC cells & in vivo tumor model	[92]

## 5. Redox Regulation of TGF-β/SMAD Pathways

Members of the TGF-β (transforming growth factor beta) superfamily orchestrate a wide array of cellular processes—ranging from proliferation, adhesion, and migration to differentiation and apoptosis. Dysregulated TGF-β signaling underlies numerous pathologies, including cancer, fibrosis, and wound-healing disorders. Ligand binding induces assembly of type II and type I serine/threonine kinase receptors into receptor complexes, triggering phosphorylation of receptor-regulated SMADs. Once phosphorylated, SMAD2/3 pair with the common mediator SMAD4 and translocate to the nucleus, where they directly regulate target-gene transcription. Despite its apparent simplicity, this SMAD-centric cascade generates highly nuanced, context-dependent transcriptional programs [97]. Although TGF-β signals through receptor-mediated SMAD phosphorylation, its outcomes are finely tuned by the cell’s redox components.

**Figure 4:**
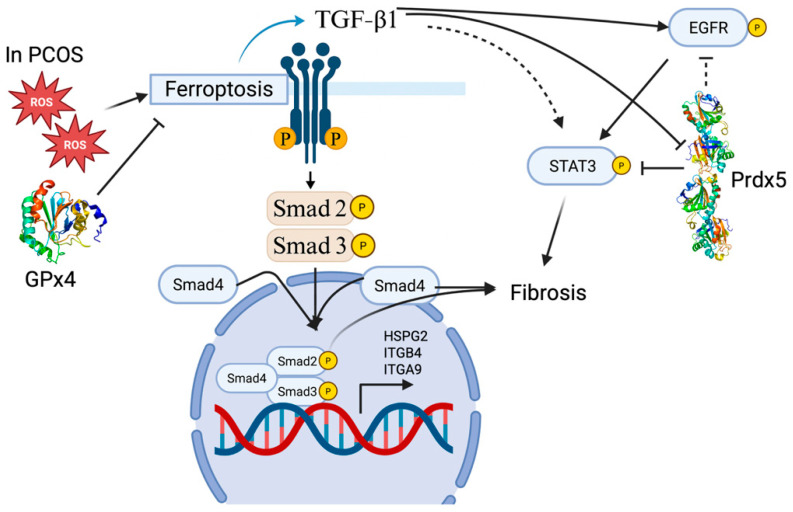
Model of TGF-β–mediated fibrosis regulated by redox enzymes. In PCOS, reduced GPx4 promotes ROS accumulation, ferroptosis, and TGF-β1/SMAD2/3-driven fibrosis. In kidney fibrosis, TGF-β1 also activates EGFR/STAT3 signaling. Prx5 inhibits STAT3 activation via direct binding, but has no effect on SMAD2/3. Figure prepared with BioRender.

A wide range of studies now show that changing a cell’s internal redox balance—especially through molecules like glutathione and glutathione peroxidases—can profoundly alter how TGF-β/SMAD signals drive fibrosis, cell growth, or survival. For example, in high-glucose models of pancreatic fibrosis, excess sugar triggers a vicious ROS–TGF-β1–SMAD3/4 loop in pancreatic stellate cells, but exogenous glutathione breaks this loop, prevents SMAD3 activation, and stops the cells from becoming activated, proliferating, or migrating [98]. In other tissues, similar redox–SMAD interactions are at play: in ovarian cancer, GPx3 helps maintain levels of GDF15 (a TGF-β family member) and supports tumor growth, whereas knocking down GPx3 in ID8 cells lowers GDF15, delays ascites onset, and markedly reduces omental tumors in mice [99]. In the endometrium of women with polycystic ovary syndrome (PCOS), too little GPx4 leads to a TGF-β1/SMAD2/3-driven buildup of extracellular matrix and fibrosis (Figure 4) [100]. Also, in blood vessels chronically exposed to TGF-β1, endothelium-dependent relaxation is lost but can be rescued by inhibiting NADPH oxidase with apocynin or by supplying extra SOD; long-term TGF-β1 overexpression leads to hypertension, cardiac remodeling, and faster atherosclerosis, all of which improve when ROS are blocked or scavenged [101]. Furthermore, in severe Plasmodium vivax malaria, high plasma heme raises SOD1 levels and lowers circulating TGF-β; adding heme to human peripheral blood mononuclear cells increases SOD1 secretion and activity while suppressing TGF-β release, and knocking down SOD1 restores TGF-β—consistent with SOD1-derived H_2_O_2_ mediating suppression; the effect was largely independent of CD14 (cluster of differentiation 14) [102].

Building on how global redox systems tune TGF-β/SMAD outputs, we next turn to peroxiredoxins—and by extension the thioredoxin axis—as direct modulators of TGF-β activity. In lens epithelial cells from Prx6^−^/^−^ mice, unchecked ROS accumulation drives up TGF-β1 mRNA, protein, and secretion, whereas treating these cells with SOD or re-expressing Prx6 restores the redox balance and prevents TGF-β1 activation [103]. Conversely, adding TGF-β1 to wild-type cells sparks ROS burst, which again is blocked by Prx6, confirming that peroxiredoxin-dependent scavenging restrains both ROS and TGF-β1 feed-forward loops [103]. Sun et al. extended this concept to immune cells, showing that recombinant tapeworm thioredoxin peroxidase (TPx) enriches the TGF-β signaling pathway in Jurkat T cells and upregulates TGF-β receptor type 1, highlighting thioredoxin’s capacity to reshape TGF-β responses via redox-sensitive transcriptional programs [104]. Similarly, in a rat UUO fibrosis model, Prx5 levels fall as fibrosis progresses, and in NRK49F fibroblasts, wild-type—but not enzymatically inactive—Prx5 overexpression blocks TGF-β–induced fibronectin and alpha–smooth muscle actin via inhibition of STAT3 (Signal Transducer and Activator of Transcription 3) phosphorylation without altering SMAD2/3 (Figure 4) [105]. Furthermore, Prx5-silenced UUO (unilateral ureteral obstruction) mice and 209/MDCT cells expressing C48S mutant Prx5 reveal that loss of Prx5’s peroxidatic cysteine biases TGF-β responses toward the EGFR/STAT3 axis, while only wild-type Prx5 suppresses EGFR-Y1068 and Stat3-Y705 phosphorylation and limits renal fibrosis (Figure 4) [106]. Beyond peroxiredoxins, other redox systems also feed back into TGF-β control—most notably the thioredoxin axis.

The thioredoxin system similarly gates TGF-β signaling at multiple nodes. First, Trx directly binds MPK38 (p38-regulated/activated protein kinase) via Cys32/Cys35 and is phosphorylated at Thr76, a modification required for Trx to block both MPK38-driven TGF-β transcriptional activity and TGF-β–induced apoptosis; inhibiting thioredoxin reductase disassembles the Trx–MPK38 complex, stabilizing MPK38, amplifying TGF-β signaling in cells, and in ASK1 (apoptosis signal-regulating kinase 1)-, SMAD3-, or p53-deficient fibroblasts, Trx overexpression prevents both MPK38-mediated and canonical SMAD activation, highlighting this redox-dependent interaction as a key brake on TGF-β pathways [107]. In the liver, TrxR3^−^/^−^ mice exposed to nickel exhibit elevated PERK (PKR-like endoplasmic reticulum kinase) and ROS that drive up TGF-β1 mRNA/protein and worsen fibrosis, all of which is reversed by melatonin’s antioxidant effect—demonstrating that TrxR3 maintains redox balance to restrain PERK-mediated TGF-β1 induction and fibrosis [108]. Lastly, in a normal murine mammary gland epithelial cell line, TGF-β1 triggers sequential cytosolic and mitochondrial ROS bursts that compromise mitochondrial membrane potential; expressing mitochondrial Trx or adding chemical antioxidants blocks TGF-β1–induced fibronectin, HMGA2 (high-mobility group AT-hook 2), and downstream Snail/Slug without altering SMAD3, indicating mitochondrial redox control as a critical determinant of TGF-β-driven EMT and epithelial plasticity [109]. Antioxidant systems—from glutathione peroxidases to peroxiredoxins and thioredoxin—tune TGF-β/SMAD signaling by controlling ROS and redox-sensitive targets, leading to logically consider them as therapeutic nodes.

## 6. Redox Regulation of NF-κB Signaling: Mechanisms and Therapeutic Insights

NF-κB (nuclear factor kappa-light-chain-enhancer of activated B cells) is an inducible family of transcription factors at the heart of innate and adaptive immune responses, relaying signals from cytokine receptors, pattern-recognition receptors, and TNF-receptor superfamily members through both canonical and noncanonical pathways. Once activated, NF-κB drives expression of pro-inflammatory mediators (cytokines, chemokines), controls immune-cell survival, activation, and differentiation, and primes inflammasome assembly [110]. Because ROS—particularly H_2_O_2_—serve as critical second messengers in many of these activation pathways, cells have evolved antioxidant systems to fine-tune NF-κB activity (Table 4). Previously, Schreck et al. established that reactive oxygen intermediates act as widely used second messengers that activate NF-κB and are sufficient to trigger HIV-1 (human immunodeficiency virus type 1) long terminal repeat-dependent expression and viral replication in T cells [111]. Also, Anderson et al. showed that NF-κB activation comprises separable oxidant-initiated pathways and a common, redox-regulated step that requires protein tyrosine phosphorylation [112]. In the sections that follow, we explore how enhancing H_2_O_2_ clearance via catalase—whether by genetic overexpression, pharmacological activation, or compartment-specific manipulation—acts as a redox brake on NF-κB signaling.

### 6.1. Catalase-Centered Redox Strategies for Modulating NF-κB Signaling

Catalase sits at the nexus of redox and inflammatory signaling by specifically decomposing H_2_O_2_, a key second messenger in NF-κB activation.

#### 6.1.1. Genetic Catalase Overexpression as a Redox Brake on NF-κB Activation

In the diabetic heart, both Wang et al. and Cong et al. used STZ to induce type 1 diabetes in mice carrying cardiomyocyte-specific catalase transgenes. Wang and colleagues showed that boosting catalase activity in the diabetic myocardium sharply reduces ROS levels, prevents nuclear accumulation of the NF-κB p65 subunit, and blunts autophagy by lowering Beclin-1 and LC3-II expression—effects that are fully recapitulated by the NF-κB inhibitor [113]. Cong et al. previously reported that catalase overexpression also curtails reactive nitrogen species, blocks IκBα (inhibitor of κB alpha) phosphorylation and p65 nuclear translocation, and thereby suppresses cytokines (IL(interleukin)-6, IL-1β), plasminogen activator inhibitor-1, and iNOS (inducible nitric oxide synthase). By preventing 3-nitrotyrosine modifications on key metabolic enzymes (α-KGD (α-ketoglutarate dehydrogenase) and ATP synthase), catalase preserves both enzymatic activity and cardiac function in the face of diabetic stress [114]. Beyond the heart, in a cancer-cell context, Lüpertz et al. showed that TNF-α–stimulated HCT-116, Caco-2, and MCF-7 cells downregulate endogenous catalase, allowing H_2_O_2_ to accumulate and drive prolonged NF-κB p65 nuclear localization. Forced catalase overexpression in MCF-7 cells restores redox balance, converting TNF-α’s sustained NF-κB activation into a brief, transient pulse and, intriguingly, sensitizing cells to apoptosis [115]. Building on the proof-of-principle that boosting catalase expression can reset NF-κB dynamics in disease-relevant models, researchers have turned to more tractable pharmacological approaches. In the next section, we examine how small-molecule activators, enzyme mimetics, and nanodelivery systems harness catalytic antioxidant activity to clear H_2_O_2_ and blunt NF-κB-driven inflammation.

#### 6.1.2. Catalytic Antioxidant Strategies to Inhibit NF-κB Activation

In murine RAW264.7 cells, lotus seed protein isolate (LSPI) boosted catalase activity to clear H_2_O_2_, blocking NF-κB p65 nuclear translocation and MAPK phosphorylation and sharply reducing iNOS, COX-2 (cyclooxygenase-2), TNF-α, IL-6, and IL-1β expression [116]. Similarly, mangiferin, a natural bioactive compound [117], directly binds and activates catalase in human U-937 macrophages and HepG2 hepatocytes, preventing IKK (IκB kinase)-mediated IκBα degradation and NF-κB p65 translocation in response to TNF-α, LPS (lipopolysaccharide), PMA (phorbol 12-myristate 13-acetate), or exogenous H_2_O_2_ [118]. Also, in breast cancer cells, the dual SOD/catalase mimetic EUK-134—but not an SOD-only mimic—clears both superoxide and H_2_O_2_ to inhibit TNF-α–induced NF-κB activation, suppress tumorigenic behaviors, and induce cell-cycle arrest and apoptosis [119]. In vivo, inhaled rhCatalse partially restores catalase and SOD activity in H1N1 (influenza A virus subtype H1N1)-infected lungs and downregulates TLR (Toll-like receptor)-4, TLR-7 and NF-κB p65 gene expression, indicating transcriptional suppression of the TLR–NF-κB pathway [120]. Catalase-loaded nanogels engineered for inhalation efficiently scavenge ROS in the airways, normalize the balance between phosphorylated and total NF-κB p65, inhibit the NLRP3 inflammasome, and reduce both inflammatory cytokines and bacterial burden in a neutrophil-driven asthma model [121]. Also, in rat heart muscle cells, the peptide angiotensin-(1–7) binds to the MAS GPCR (G-protein-coupled receptor), triggering activation of the transcription factor FOXO3 (Forkhead box O3), which in turn up-regulates catalase and SOD1, lowering ROS levels and preventing NF-κB-mediated hypertrophic growth of the cardiomyocytes [122]. It is noteworthy that in certain contexts, altering catalase activity or localization can have unexpected, compartment-specific effects on NF-κB signaling.

#### 6.1.3. Localized Catalase Effects on NF-κB Signaling

Han et al. found that mice overexpressing mitochondrial-targeted catalase (mCAT) develop stronger LPS-induced lung inflammation: by stripping H_2_O_2_ from the cytosol, mCAT raises NADH/NAD^+^ ratios and ATP production in macrophages, which paradoxically amplifies NF-κB activation via metabolic signaling [123]. In contrast, Mu et al. showed that inhibiting catalase with 3-AT (3-amino-1,2,4-triazole) drives a build-up of peroxisomal ROS, leading to lipid peroxidation and formation of 4-HNE–IκBα adducts that lock IκBα in an unphosphorylatable state—thereby blocking NF-κB nuclear translocation and downstream cytokine expression [124]. Together, these studies indicate that both redox compartmentalization and metabolic flux can flip NF-κB responses. Building on this modulation of NF-κB by catalase, application of exogenous catalase further indicates that H_2_O_2_ scavenging outside the cell can paradoxically trigger PI3K-dependent NF-κB–mediated inflammation. In BV2 microglial cells and RAW264.7 macrophages, adding catalase exogenously unexpectedly drives pro-inflammatory gene expression. Catalase rapidly degrades IκBα, activates NF-κB promoter activity, and engages PI3K/AKT, p70S6K, and JNK (c-Jun N-terminal kinase) signaling to boost COX-2 and iNOS mRNA and protein; blocking PI3K halts both kinase activation and NF-κB–dependent transcription [125]. Also, catalase induces iNOS by enhancing both transcription and mRNA stability; inhibiting NF-κB or PI3K abolishes this effect, and the response is specific to macrophage-lineage cells [126]. A parallel study shows that catalase up-regulates COX-2 via NF-κB and PI3K, without involvement of ERKs, p38s, or JNKs in mRNA stabilization [127]. Together, these findings reveal that, beyond breaking down H_2_O_2_, exogenous catalase can orchestrate a PI3K-dependent NF-κB cascade to promote inflammatory mediator expression in myeloid cells.

### 6.2. Glutathione Peroxidases in NF-κB Regulation

Genetic and isoform-focused studies have revealed how individual glutathione peroxidases act as bespoke brakes on NF-κB activation in diverse contexts. In the next section, we turn to isoform-specific and genetic approaches that have shown the distinct roles of some GPx family members in tempering NF-κB activation

#### 6.2.1. Isoform-Specific and Genetic Dissection of GPx-Mediated NF-κB Regulation

Crack et al. showed that mice lacking GPx1 suffer larger infarcts after cerebral ischemia–reperfusion because elevated ROS drives serine-536 phosphorylation of NF-κB p65 and enhanced p50/p65 DNA binding—effects that a broad-spectrum NF-κB inhibitor rescues [128]. Brigelius-Flöhé highlights that of the five mammalian GPx isoforms, although they share glutathione-dependent peroxide reduction, phospholipid-hydroperoxide GPx (PHGPx, or GPx4) suppresses cytokine- and stress-induced NF-κB activation, while gastrointestinal GPx (GPx2) is transcriptionally linked to Nrf2 and protects the gut from inflammation [129]. In human Caco-2 cells, Gong et al. found that selenium deprivation lowers GPx1 and amplifies TNF-α–triggered NF-κB and IL-8 induction, whereas targeted GPx1 or GPx4 knock-downs show that GPx1 is required for full TNF-α–induced NF-κB/IL-8 (knockdown lowers the response), while GPx4 governs flagellin-mediated responses [130]. Peng et al. uncovered GPx7’s role in Barrett’s esophagus: reconstituting GPx7 in bile-salt–treated or TNF-α–stimulated esophageal cells inhibits p65 phosphorylation and cytokine/chemokine up-regulation, and clinical samples show inverse correlations between GPx7 levels and pro-inflammatory transcripts, implicating GPx7 loss in tumorigenesis [131]. A follow-up study by the same group demonstrated that GPx7 suppresses NF-κB independently of its antioxidant activity by accelerating degradation of TNFR1 (tumor necrosis factor receptor 1) and TRAF2 (TNF receptor–associated factor 2), thereby disassembling upstream signaling complexes and preventing p65 activation [132]. Building on these mechanistic insights into GPx isoforms’ roles in NF-κB suppression, the next section explores pharmacological and nutritional strategies to harness and enhance GPx activity for therapeutic inhibition of NF-κB.

#### 6.2.2. Enhancement of GPx Activity to Suppress NF-κB Signaling

A variety of translational approaches have enhanced glutathione peroxidase activation to blunt NF-κB-driven pathologies. Li et al. discovered a small-molecule allosteric activator, compound 102, that doubles GPx4 activity, shifts arachidonic acid metabolism toward 12- and 15-HETEs (hydroxyeicosatetraenoic acids) (which can be further metabolized into anti-inflammatory mediators), reduces ROS, and dose-dependently inhibits TNF-α–induced NF-κB reporter activity while protecting against ferroptosis [133]. In a gene therapy strategy, Sharma et al. used an adenoviral vector to restore GPx1 in GPx1^−^/^−^ mice, which prevented methamphetamine-induced NF-κB activation, preserved striatal dopamine levels and tyrosine hydroxylase function, and improved motor behavior—effects fully recapitulated by the NF-κB inhibitor pyrrolidine dithiocarbamate [134]. Also, Wang et al. showed that cochlear neurons exposed to peroxynitrite lose GPx1 and activate NF-κB, but pretreatment with ebselen, a GPx1 mimic, restores enzyme levels, reduces lipid peroxidation, and rescues neuronal survival by attenuating p65 translocation [135]. Dietary Maca polysaccharides bolster hepatic GPx and SOD, mitigate aflatoxin B_1_–induced liver injury, reprogram AhR/STAT3 in macrophages, and suppress NF-κB p65 activation and pro-inflammatory cytokines [136]. Lately, Yang et al. engineered mannose-functionalized selenium nanoparticles in a colon-targeted hydrogel to up-regulate GPx1–4 in intestinal epithelial cells, block dextran sulfate sodium DSS-induced NF-κB p65 activation, and alleviate ulcerative colitis in mice [137]. Collectively, these studies demonstrate the broad therapeutic potential of enhancing GPx activity to blunt NF-κB-driven inflammation. Conversely, peroxidase-generated halogenants can activate NF-κB, driving inflammatory and vascular responses.

### 6.3. Peroxidase-Driven NF-κB Modulation

In asthma, airway epithelia convert thiocyanate and H_2_O_2_ into hypothiocyanite (OSCN^−^) via the pendrin/dual oxidase/peroxidase axis; low OSCN^−^ doses activate NF-κB via protein kinase A to induce chemokines, while high levels trigger necrosis and IL-33 release, amplifying type-2 inflammation—a pathway attenuated by heme peroxidase inhibitors (antithyroid agents) [138]. Previously, it was shown that endothelial cells exposed to OSCN^−^ markedly up-regulate endothelial adhesion molecules and bind neutrophils or eosinophils via an NF-κB–dependent mechanism, and intraperitoneal OSCN^−^ drives neutrophil extravasation in vivo [139]. Furthermore, OSCN^−^ potently induces tissue factor in HUVECs (human umbilical vein endothelial cells) by activating NF-κB (p65/p50 and p65/c-Rel) heterodimers and ERK1/2–Egr-1 (early growth response 1) signaling; blocking NF-κB or ERK1/2 abrogates TF (tissue factor) up-regulation, suggesting that peroxidase-derived OSCN^−^ can provoke a prothrombotic endothelial state in hypereosinophilic conditions [140]. Also, in human IMR-32 neuroblastoma cells, eosinophil peroxidase binds to cell-surface glycosaminoglycans to trigger ERK1/2 phosphorylation and NF-κB activation, which drives choline acetyltransferase and VAChT (vesicular acetylcholine transporter) expression to promote cholinergic plasticity [141]. By contrast, the thioredoxin-dependent peroxidase AOE372 acts intracellularly in HeLa cells: it rapidly reduces H_2_O_2_ and prevents IκBα phosphorylation and NF-κB (p65/p50) nuclear accumulation in response to TNF-α, TPA (12-O-tetradecanoylphorbol-13-acetate), or HIV-1 Tat, thereby suppressing NF-κB-driven gene expression [142]. Building on peroxidase-mediated fine-tuning of NF-κB, superoxide dismutases likewise exert context-dependent control over NF-κB signaling—suppressing or promoting inflammation depending on cellular and oxidative conditions.

### 6.4. Context-Dependent Modulation of NF-κB Signaling by SOD

In the skin, EC-SOD (extracellular superoxide dismutase) blocks NF-κB activation by preventing phosphorylation of its p65 subunit. This event stops p65 from moving into the nucleus and switching on inflammatory genes. By scavenging extracellular superoxide, EC-SOD reduces the ROS-driven activation of upstream kinases (like protein kinase C) that would normally phosphorylate NF-κB p65, so fewer pro-inflammatory mediators (e.g., COX-2, iNOS) are produced [143]. By contrast, in pancreatic cancer cells, Li et al. found that adding SOD raises intracellular H_2_O_2_, triggering ERK and NF-κB activation to drive EMT, invasion, and wound closure—effects that are reversed by catalase or ERK inhibition [144]. Also, in a murine Ehrlich ascites carcinoma model, Abu-Zeid et al. observed that nicotine down-regulates SOD1/2, catalase, and GPx1/2 and up-regulates NF-κB, worsening liver oxidative damage and inflammation [145]. These studies show that SOD’s impact on NF-κB is highly context-dependent—protecting against inflammation and angiogenesis in healthy tissues while potentially promoting EMT, invasion, and oxidative injury under pathological conditions. In a similar vein, the thioredoxin system represents another major redox axis that fine-tunes NF-κB transactivation through complementary cytosolic, mitochondrial, and post-translational mechanisms.

### 6.5. The Thioredoxin Network in NF-κB Regulation

Heilman et al. established that TrxR1 is indispensable for NF-κB-driven gene expression: inhibiting TrxR1 pharmacologically or by siRNA markedly inhibits both NF-κB reporter activity and endogenous IκB-α mRNA induction without altering IκB-α degradation, p50/p65 nuclear translocation, or DNA binding. Crucially, neither the redox state of NF-κB subunits nor Trx1 oxidation explains this block; rather, TrxR1 governs NF-κB’s transactivation potential via post-translational mechanisms [146]. Previously, Sakurai et al. showed that TNF-α up-regulates TrxR1 (but not TrxR2/3) in endothelial and COS7 cells and that TrxR1 overexpression amplifies TNF-α–triggered NF-κB reporter activity and target-gene expression (e.g., E-selectin, COX-2). Inhibiting TrxR1 suppresses these effects [147]. In addition, while TrxR1 enhances NF-κB transactivation downstream of DNA binding via redox-sensitive p65 phosphorylation, mitochondrial Trx2 acts upstream by clearing ROS to prevent p65/p50 nuclear translocation and restrain NF-κB activation. In TNF-α–stimulated cells, Trx2—but not cytosolic Trx1—overexpression blunts ROS accumulation, prevents p65/p50 nuclear translocation, and suppresses NF-κB reporter activity, demonstrating that mitochondrial ROS detoxification is sufficient to inhibit cytokine-driven signaling [148]. In macrophages, restoring Trx2 via lentiviral delivery markedly reduces IL-6 and TNF-α production by blocking p65 phosphorylation, nuclear translocation, and upstream MAPK activation; in septic mice, Trx2 overexpression likewise improves survival and limits organ injury [149]. Complementing these findings in adipocytes, H_2_O_2_ exposure suppresses Trx2 expression, depletes ATP and antioxidant capacity, and triggers reduced IκBα protein abundance, NF-κB phosphorylation, and pro-inflammatory cytokine expression—effects recapitulated by Trx2 knockdown and reversed by Trx2 overexpression or NAC (N-acetylcysteine) [150].

Building on TrxR1’s cytosolic activation and Trx2’s mitochondrial inhibition of NF-κB, thioredoxin-family modulators further fine-tune NF-κB activity through denitrosylation (TXNIP) sequestration and subcellular trafficking. In respiratory epithelia, Trx released from TXNIP inhibition removes S-nitrosyl adducts from NF-κB p65, a step essential for p65 DNA binding and target-gene induction; blocking TrxR or knocking down Trx prevents p65 denitrosylation and downstream cytokine expression in vitro and in injured lungs in vivo [151]. In CRC, the thioredoxin-like enzyme Txl-2b (thioredoxin-like protein 2b) is overexpressed in most tumors and drives IκBα and p65 phosphorylation, nuclear p65 accumulation, and NF-κB–dependent expression of Cyclin D1, Bcl-2 family proteins, and Survivin; silencing Txl-2b reverses these effects, impeding proliferation and chemoresistance [152]. In airway epithelia, the nuclear translocation of Trx sustains NF-κB DNA binding and pro-inflammatory cytokine production; blocking Trx’s nuclear entry with cHCEU attenuates NF-κB DNA binding/activation even when Nrf2 and Trx levels rise [153]. Furthermore, in neurons, the lignan Schisanhenol protects against MPP^+^ (1-methyl-4-phenylpyridinium) toxicity by up-regulating Trx1, thereby preventing ASK1–p38 activation, IκBα degradation, and NF-κB nuclear translocation to block apoptosis [154]. Recently, it was found that UV-B-irradiated squamous-cell carcinoma cells exploit Thioredoxin domain-containing protein 9 (TXNDC9) to facilitate IκBα phosphorylation and p65 translocation, promoting survival; knocking down TXNDC9 restores apoptosis and impairs NF-κB activation [155]. Overall, thioredoxin-family proteins fine-tune NF-κB activity through different mechanisms, underscoring their versatility as regulators of inflammation and cell survival. Building on the thioredoxin network’s redox regulation of NF-κB, antioxidant enzymes like peroxiredoxins also modulate developmental pathways such as Hedgehog to sustain cancer stem cell properties and tumor progression.

**Table 4:** **Summary of redox components controlling NF-κB signaling:** This table lists redox components—overexpression, activation, inhibition, or mimetics of catalase, SOD, GPx, and thioredoxin systems—applied across various cellular and animal models. It details their impacts on NF-κB signaling, including changes in ROS/RNS levels, IκBα phosphorylation, p65 translocation, cytokine production, and associated phenotypic outcomes.

Intervention	Model/System	NF-κB Read-Out	Ref.
Cardiomyocyte-specific catalase overexpression	STZ-induced type 1 diabetic mouse heart	↓ ROS levels; ↓ nuclear NF-κB p65 translocation; ↓ Beclin-1 & LC3-II expression (blunted autophagy)	[113]
Cardiomyocyte-specific catalase overexpression	STZ-induced type 1 diabetic mouse heart	↓ reactive nitrogen species; blocked IκBα phosphorylation & p65 nuclear translocation; ↓ IL-6, IL-1β, myeloperoxidase, iNOS; prevented 3-nitrotyrosine modifications on α-KGD & ATP synthase, preserving enzyme activity and cardiac function	[114]
Forced catalase overexpression	TNF-α–stimulated HepG2, Caco-2, and MCF-7 cancer cells (MCF-7 overexpression)	Converts prolonged p65 nuclear localization into a brief, transient pulse; sensitizes cells to apoptosis	[115]
Lotus seed protein isolate–mediated catalase activation	Murine RAW264.7 macrophages	Blocked NF-κB p65 nuclear translocation & MAPK phosphorylation; ↓ iNOS, COX-2, TNF-α, IL-6, IL-1β expression	[116]
Mangiferin-mediated catalase activation	Human U-937 macrophages & HepG2 hepatocytes	Prevented IKK-mediated IκBα degradation & NF-κB p65 nuclear translocation in response to TNF-α, LPS, PMA, or H_2_O_2_	[118]
EUK-134 dual SOD/catalase mimetic	TNF-α–stimulated breast cancer cells	Clears superoxide & H_2_O_2_; inhibits TNF-α–induced NF-κB p65 activation; suppresses tumorigenic behaviors; induces cell-cycle arrest & apoptosis	[119]
Inhaled recombinant catalase (rhCatalase)	H1N1-infected mouse lungs (in vivo)	↓ TLR-4, ↓ TLR-7, ↓ NF-κB p65 gene expression (transcriptional suppression of the TLR–NF-κB pathway)	[120]
Catalase-loaded inhalable nanogels	Neutrophil-driven asthma model (airways)	Normalized phosphorylated/total p65 ratio; inhibited NLRP3 inflammasome; reduced inflammatory cytokines & bacterial burden	[121]
Mitochondrial-targeted catalase overexpression (mCAT)	LPS-induced lung inflammation in mice	↑ NF-κB activation via metabolic signaling (raised NADH/NAD^+^ ratio and ATP production)	[123]
Catalase inhibition with 3-AT	Peroxisomal ROS accumulation model	Blocked NF-κB nuclear translocation & downstream cytokine expression via 4-HNE–IκBα adducts	[124]
Exogenous catalase addition	BV2 microglia & RAW264.7 macrophages	Rapid IκBα degradation; NF-κB promoter activation; engagement of PI3K/AKT, p70S6K & JNK signaling; ↑ COX-2 & iNOS mRNA/protein (PI3K blockade halts effect; p70S6K & JNK inhibitors distinguish translational vs. transcriptional control)	[125]
Exogenous catalase addition	Macrophage-lineage cells	Enhanced iNOS transcription & mRNA stability (abolished by NF-κB or PI3K inhibition); up-regulated COX-2 via NF-κB & PI3K (independent of ERK, p38, or JNK for mRNA stabilization)	[126,127]
GPx1 knockout	Mice after cerebral ischemia–reperfusion	↑ p65 Ser536 phosphorylation; ↑ p50/p65 DNA binding (effects reversed by broad-spectrum NF-κB inhibitor)	[128]
GPx4 & GPx2 isoform activity	Mammalian contexts	GPx4 specifically suppresses cytokine- and stress-induced NF-κB activation; GPx2 (via Nrf2 linkage) helps protect the gut from NF-κB-driven inflammation	[129]
Selenium deprivation & targeted GPx1/GPx4 knock-down	Human Caco-2 cells	Selenium deprivation ↑ TNF-α–triggered NF-κB activation & IL-8 induction; GPx1 knock-down removes restraint on cytokine-driven NF-κB; GPx4 knock-down enhances flagellin-mediated NF-κB	[130]
GPx7 reconstitution	Bile-salt-treated or TNF-α-stimulated Barrett’s esophagus esophageal cells	Inhibits NF-κB p65 phosphorylation and downstream cytokine/chemokine up-regulation; clinical samples show inverse correlation between GPx7 levels and pro-inflammatory transcripts	[131]
GPx7 overexpression (antioxidant-independent mechanism)	Barrett’s esophagus esophageal cells	Accelerated proteasomal degradation of TNFR1 & TRAF2; disassembly of upstream signaling complexes; prevented p65 activation	[132]
Compound 102 (small-molecule GPx4 activator)	In vitro TNF-α–stimulated cellular NF-κB reporter assay	↓ TNF-α–induced NF-κB reporter activity; ↑ anti-inflammatory HETEs; ↓ ROS; protection against ferroptosis	[133]
Adenoviral GPx1 restoration	GPx1^−^/^−^ mice exposed to methamphetamine	Prevented meth-induced NF-κB activation; preserved striatal dopamine & tyrosine hydroxylase levels; improved motor behavior	[134]
Ebselen pretreatment (GPx1 mimic)	Cochlear neurons exposed to peroxynitrite	Restored GPx1 levels; ↓ lipid peroxidation; attenuated p65 nuclear translocation; rescued neuronal survival	[135]
EC-SOD (extracellular SOD)	Skin (keratinocytes/mouse skin)	Prevented p65 phosphorylation & nuclear translocation; ↓ COX-2 and iNOS expression	[143]
Exogenous SOD addition	Pancreatic cancer cells	↑ Intracellular H_2_O_2_; activated ERK & NF-κB driving EMT, invasion, wound closure (effects reversed by catalase or ERK inhibition)	[144]
TrxR1 inhibition (curcumin, CDNB, or siRNA)	Cultured cells (NF-κB reporter assay; endogenous IκB-α mRNA)	Inhibits NF-κB reporter activity & IκB-α mRNA induction; no effect on IκB-α degradation, p50/p65 nuclear translocation, or DNA binding—indicating a block at Ser536 phosphorylation–dependent transactivation	[146]
Mitochondrial Trx2 overexpression	TNF-α-stimulated cells	↓ ROS accumulation; prevented p65/p50 nuclear translocation; suppressed NF-κB reporter activity	[148]
Trx2 restoration via lentiviral delivery	Macrophages; septic mice	↓ IL-6 & TNF-α production by blocking p65 phosphorylation, nuclear translocation & upstream MAPK activation; improved survival and reduced organ injury in vivo	[149]
H_2_O_2_ exposure (±TRX2 manipulation)	Adipocytes	H_2_O_2_: ↓ TRX2, ↓ ATP & antioxidant capacity → IκBα degradation, NF-κB phosphorylation & ↑ pro-inflammatory cytokines; reversed by TRX2 OE or NAC	[150]
Trx release from TXNIP inhibition	Respiratory epithelial cells (in vitro & injured lungs in vivo)	Removal of S-nitrosyl adducts from p65 enabling DNA binding and target-gene induction; blocking TrxR or Trx knockdown prevents p65 denitrosylation and downstream cytokine expression	[151]
Txl-2b overexpression	Colorectal cancer cells	↑ IκBα & p65 phosphorylation; ↑ nuclear p65 accumulation; ↑ NF-κB–dependent Cyclin D1, Bcl-2 family, and Survivin expression; silencing Txl-2b reverses these effects, reducing proliferation and chemoresistance	[152]
Blockade of Trx nuclear entry with cHCEU	Airway epithelial cells	Abolished NF-κB DNA binding and pro-inflammatory cytokine production despite elevated Nrf2 and Trx levels	[153]
Schisanhenol-induced up-regulation of Trx1	Neurons exposed to MPP^+^ toxicity	Prevented ASK1–p38 activation, IκBα degradation, and NF-κB nuclear translocation, thereby blocking apoptosis	[154]
TXNDC9 knockdown in UV-B–irradiated squamous-cell carcinoma cells	UV-B–irradiated human squamous-cell carcinoma cells	Reduced IκBα phosphorylation and p65 translocation, restoring apoptosis and impairing NF-κB-driven survival signaling	[155]

## 7. Redox Modulation of Hedgehog, Notch, and G-Protein-Coupled Receptor Signaling

The Hedgehog (Hh) pathway governs tissue development by a straightforward “on/off” mechanism: secreted Hh ligands bind the 12-transmembrane receptor Patched, relieving its inhibition of Smoothened (SMO). Once free, SMO activates Gli (zinc-finger transcription factors), which translocate into the nucleus and switch on genes that drive cell proliferation, differentiation, and patterning [156]. In CRC, the thiol-dependent Prx2 has emerged as a key supporter of CSCs. When Prx2 expression is knocked down using shRNA, CSCs show reduced self-renewal and sphere formation, and they become more prone to apoptosis upon 5-fluorouracil treatment. Conversely, Prx2 overexpression restores these stem-like features and maintains markers such as CD44, CD133, and Nanog —actions mediated through regulation of SMO and Gli1 in the Hh pathway. In mouse xenografts, Prx2 depletion leads to markedly smaller tumors, suggesting its role in maintaining colon CSC growth via the Hh/Gli1 axis [157]. A similar redox-driven mechanism operates in NSCLC: Prx2 promotes lung CSC traits—including self-renewal, migration, invasion, and angiogenesis—by keeping ROS in check. Silencing Prx2 raises ROS levels and induces CSC apoptosis, while overexpression rescues these properties. Importantly, Prx2 supports not only Hh (Hedgehog)/Gli1) signaling but also Notch1/Hes1 (Hairy and enhancer of split-1) and Wnt/β-catenin pathways; targeting Prx2 via miR-122 disrupts this network and suppresses CSC growth both in vitro and in vivo [90]. Beyond cancer, PRX6 modulates metabolic signaling in nonalcoholic fatty liver disease. In transgenic mice overexpressing Prx6—and in HepG2 hepatocytes—this enzyme preserves mitochondrial integrity and lowers ROS, thereby suppressing Notch signaling. As a result, lipid accumulation and metabolic dysfunction are prevented [158].

Redox enzymes also directly influence GPCR signaling. Under normoxia in prostate cancer cells, SOD1 binds the chemokine receptor CXCR4 (chemokine receptor type 4) to enhance Gα_i_-dependent Akt phosphorylation and promote survival and migration—an effect diminished under hypoxia. Likewise, upon JNK activation, Prx6 associates with opioid receptor–Gα_i_ complexes: its phospholipase A_2_ activity generates ROS that trigger oxidative depalmitoylation of Gα_i_, dampening receptor signaling and contributing to opioid tolerance. Inhibiting Prx6 or scavenging ROS restores normal receptor function, suggesting novel redox-dependent control of GPCR activity [159,160].

## 8. Future Directions

Systematic investigation into redox-sensitive modifications and diverse signaling pathways—including Wnt, Notch, TGF-β, PI3K-Akt, and mTOR—is essential for developing targeted therapies across diverse pathological contexts [1]. Specifically, studies should clarify Trx1-mediated redox mechanisms in Wnt/β-catenin signaling during osteogenesis and assess their broader relevance to tissue repair [80]. Additionally, examining Prx2’s role in β-catenin stabilization and Wnt activation may offer therapeutic insights into hepatocellular carcinoma and related cancers [92]. Also, exploring how redox agents like hyperforin induce ferroptosis by disrupting iron homeostasis and antioxidant defenses (GPx4, SLC7A11 (cystine/glutamate antiporter subunit), ferritin) and interact with melanoma pathways (HMOX1–BACH1 (BTB and CNC homology 1), MAPK, EMT markers) could advance treatment strategies [40]. TXNDC9’s modulation of NF-κB/p65 signaling in cutaneous squamous cell carcinoma also warrants evaluation as a therapeutic target [155]. Validation of the IGF-1–GPx1–NMD axis and enhancing selenocysteine metabolism in ovarian cyst models may sustainably restore redox homeostasis and improve oocyte quality [59]. Further, GPx3’s regulation of GDF15 expression could enhance ovarian cancer immune therapies by targeting extracellular GPx3 or GDF15 [99]. Integrated analyses of electrophile-induced RTK/PTP signaling, Txnrd1 activity, and KEAP1/NRF2 responses could identify concentration- and compartment-specific redox therapeutic opportunities [51]. Evaluating SOD1 inhibitors like ATN-224 alongside mTORC1 inhibitors in glioblastoma should prioritize identifying responsive tumor subtypes [67]. Restoring redox balance by targeting glutathione pathways or Nrf2/NF-κB interactions may mitigate oxidative injury in cholangiopathies like primary sclerosing cholangitis [96]. Furthermore, clinical evaluation of catalase-loaded nanogels (CAT-NGs) for ROS-driven respiratory diseases should also proceed to confirm efficacy and safety [121]. Clarifying dietary Maca polysaccharides’ modulation of macrophage polarization and NRF2/STAT3 pathways against aflatoxin B1 toxicity and validating Chlorella vulgaris’ protective effects on NF-κB and caspase signaling in nicotine-induced cancer represent promising redox therapeutic strategies [136,145]. In immunology, validating tapeworm-derived TPx’s role in modulating TGF-β/SMAD signaling and Th17 (T helper 17 cell subset) differentiation could elucidate immune evasion mechanisms [104]. Clarifying SMAD2/3 regulation of fibrosis-related genes via ChIP-seq (chromatin immunoprecipitation sequencing) in endometrial epithelial cells may illuminate redox–TGF-β interactions in PCOS [100]. The role of mitochondrial thioredoxin in mitochondrial function, lipolysis, and inflammation beyond NF-κB signaling should be investigated to understand metabolic disorders during transition periods in dairy cows [150]. Limited research currently addresses redox regulation of Hedgehog, Notch, and GPCR signaling pathways; exploring these interactions could significantly advance therapeutic approaches and our understanding of related disease pathogenesis. Clinical translation of redox-based therapies is actively underway, as evidenced by ongoing and completed clinical trials utilizing redox components (e.g., SOD, catalase, GPx) across multiple disease contexts. A comprehensive summary of these trials is provided in Supplementary File S1, highlighting the practical implications and clinical relevance of targeting redox pathways therapeutically.

## Figures and Tables

**Table 1:** Summary of glutathione peroxidase (GPx) isoforms (GPx1–GPx8) showing predominant localization and principal specificity. Reviewed in [10].

Enzyme	Localization	Specificity
GPx1	Cytoplasm and mitochondria	Reacts with H_2_O_2_, but not with complex lipid hydroperoxides
GPx2	Gastrointestinal system	First line of defense against oxidative stress from foods or intestinal flora
GPx3	Renal tubules	Abolishes all complex hydroperoxides, circulates in plasma as well as in cytoplasm and mitochondria
GPx4	Cytosol, mitochondria, and nucleus	The sole GPx that directly reduces complex lipid hydroperoxides
GPx5	Extracellular in epididymis	Protects sperms from hydrogen peroxide toxicity
GPx6	Embryonic and olfactory organ epithelial cells	GPx1–4 and GPx6 catalyze reduction of H_2_O_2_ or organic hydroperoxides to water or corresponding alcohols, thereby reducing their toxicity and maintaining redox balance
GPx7	Endoplasmic reticulum (ER) lumen	Utilizes H_2_O_2_ to accelerate oxidative folding process of proteins
GPx8	Enriched in mitochondria-associated membranes	Similar to GPx7 because both have low glutathione peroxidase activity

## Data Availability

No new data were generated or analyzed as part of this review.

## Supplementary Materials

Supplementary File S1. Ongoing and completed clinical studies implementing antioxidant enzymes in humans—SOD, catalase, GPx, and thioredoxin/peroxiredoxins—across diverse indications. Entries list NCT number, enzyme target, intervention, trial status, disease/indication, phase, country, year, and a registry link. The supporting information can be downloaded at: https://www.mdpi.com/article/10.3390/antiox14091142/s1.

## Abbreviations

The following abbreviations are used in this manuscript:

3-AT	3-amino-1,2,4-triazole
4E-BP1	eIF4E-binding protein 1
4-HNE	4-Hydroxynonenal
α-KGD	α-ketoglutarate dehydrogenase
ABCG2	ATP-binding cassette subfamily G member 2
AGR2	Anterior gradient 2
AhR	aryl hydrocarbon receptor
AKT	Ak strain transforming, aka. protein kinase B
APC	adenomatous polyposis coli
ASK1	apoptosis signal-regulating kinase 1
ATM	ataxia-telangiectasia mutated (kinase)
ATN-224	tetrathiomolybdate derivative
AXL	AXL receptor tyrosine kinase
AMPK	AMP-activated protein kinase
Bcl-2	B-cell lymphoma 2
BrdU	5-bromo-2′-deoxyuridine
CLL	chronic lymphocytic leukemia
COX-2	cyclooxygenase-2
CRC	colorectal cancer
CSC	cancer stem–like cell
Dvl	Dishevelled
E-selectin	endothelial-leukocyte adhesion molecule-1
EC-SOD	extracellular superoxide dismutase
EGFR	epidermal growth factor receptor
EGR1	early growth response 1
eIF4E	eukaryotic translation initiation factor 4E
EMT	epithelial–mesenchymal transition
ER	endoplasmic reticulum
ERK/ERK1/2	extracellular signal-regulated kinase (isoforms 1 and 2)
FoxA2	Forkhead box A2
GDF15	growth differentiation factor 15
GPCR	G protein-coupled receptor
GPx	glutathione peroxidases
Grx3	glutaredoxin 3
GSK-3β	glycogen synthase kinase-3β
GSTs	glutathione S-transferases
H1N1	influenza A virus subtype H1N1
H_2_O_2_	hydrogen peroxide
HCC	hepatocellular carcinoma
Hh	Hedgehog
HETEs	hydroxyeicosatetraenoic acids (e.g., 12-/15-HETE)
HIV-1	human immunodeficiency virus type 1
HMOX1	heme oxygenase-1
IGF-1R	insulin-like growth factor 1 receptor
IκBα	inhibitor of κB alpha
IKK	IκB kinase
IL	interleukin
iNOS	inducible nitric oxide synthase
JNK	c-Jun N-terminal kinase
KEAP1	Kelch-like ECH-associated protein 1
LC3	microtubule-associated protein 1 light chain 3
LPS	lipopolysaccharide
LSPI	lotus seed protein isolate
MAPK	mitogen-activated protein kinase
mCAT	mitochondrial-targeted catalase
MEK	MAPK/ERK kinase
miR-122	microRNA-122
MMP	matrix metalloproteinase
MnSOD	mitochondrial superoxide dismutase
MPK38	p38-regulated/activated protein kinase
MPP^+^	1-methyl-4-phenylpyridinium
MSCs	mesenchymal stem cells
mtDNA	mitochondrial DNA
mTORC1	mechanistic Target of Rapamycin Complex 1
NAC	N-acetylcysteine
NADH	nicotinamide adenine dinucleotide
NADPH	nicotinamide adenine dinucleotide phosphate
NF-κB	nuclear factor kappa-light-chain-enhancer of activated B cells
Nkx3.1	NK3 homeobox 1
NMD	nonsense-mediated mRNA decay
NRF	nuclear factor erythroid
NRX	nucleoredoxin
NSCLC	non-small cell lung cancer
OSCN^−^	hypothiocyanite
p70S6K	70 kDa ribosomal S6 kinase
PCOS	polycystic ovary syndrome
PDGFRβ	platelet-derived growth factor receptor-β
PDGF-BB	platelet-derived growth factor-BB
PEG-SOD	polyethylene glycol–conjugated superoxide dismutase
PEX5	peroxisomal biogenesis factor 5
PERK	PKR-like endoplasmic reticulum kinase
PI3K	phosphoinositide 3-kinase
PIN	prostatic intraepithelial neoplasia
PIP_3_	phosphatidylinositol-3,4,5-triphosphate
PP2A	protein phosphatase 2A
PMA	phorbol 12-myristate 13-acetate
Prxs	peroxiredoxins
PTP1B	protein tyrosine phosphatase 1B
PTPs	protein tyrosine phosphatases
RET	REarranged during Transfection
RNS	reactive nitrogen species
ROS	reactive oxygen species
RTKs	receptor tyrosine kinases
RXRα	retinoid X receptor alpha
sFlt-1	soluble fms-like tyrosine kinase-1, soluble VEGFR-1
siRNA	small interfering RNA
SMO	Smoothened
SODs	superoxide dismutases
STAT3	Signal Transducer and Activator of Transcription 3
STZ	streptozotocin
TCF/LEF	T-cell factor/Lymphoid enhancer factor
TCGA	The Cancer Genome Atlas
TGF-β	transforming growth factor beta
TLR-4; TLR-7	Toll-like receptor-4/-7
TNF-α	tumor necrosis factor-α
TNFR1	tumor necrosis factor receptor 1
TNKS1	tankyrase 1
TOPflash	TCF/LEF luciferase Wnt reporter assay
TPx	thioredoxin peroxidase
TRAF2	TNF receptor–associated factor 2
TRAMP	Transgenic Adenocarcinoma of Mouse Prostate
TRP14	thioredoxin-related protein 14
Trx	thioredoxin
TSC	tuberous sclerosis complex
TXL-2b	thioredoxin-like protein 2b
TXNDC9	thioredoxin domain-containing protein 9
TXNIP	thioredoxin-interacting protein
ULK1	Unc-51-like kinase 1
UUO	unilateral ureteral obstruction
UV	ultraviolet
VEGF	vascular endothelial growth factor
VEGFR1/VEGFR2	vascular endothelial growth factor receptor 1/2
Wnt	Wingless/Int

## References

[1] Li B., Ming H., Qin S., Nice E.C., Dong J., Du Z., Huang C. (2025). Redox regulation: Mechanisms, biology and therapeutic targets in diseases. Signal Transduct. Target. Ther..

[2] Thénard L.J. (1818). Observations sur des nouvelles combinaisons entre l’oxigène et divers acides. Ann. Chim. Phys..

[3] Schönbein C.F. (1863). Ueber die katalytische Wirksamkeit organischer Materien und deren Verbreitung in der Pflanzen-und Thierwelt. J. Prakt. Chem..

[4] Loew O. (1900). A new enzyme of general occurrence in organisms. Science.

[5] Zámocký M., Koller F. (1999). Understanding the structure and function of catalases: Clues from molecular evolution and in vitro mutagenesis. Prog. Biophys. Mol. Biol..

[6] Gerschman R., Gilbert D.L., Nye S.W., Dwyer P., Fenn W.O. (1954). Oxygen poisoning and x-irradiation: A mechanism in common. Science.

[7] Rotruck J.T., Pope A.l., Ganther H.E., Swanson A.B., Hafeman D.G., Hoekstra W.G. (1973). Selenium: Biochemical Role as a Component of Glutathione Peroxidase. Science.

[8] Mills G.C. (1957). Hemoglobin catabolism: I. Glutathione peroxidase, an erythrocyte enzyme which protects hemoglobin from oxidative breakdown. J. Biol. Chem..

[9] Ursini F., Maiorino M., Gregolin C. (1985). The selenoenzyme phospholipid hydroperoxide glutathione peroxidase. Biochim. Biophys. Acta (BBA)-Gen. Subj..

[10] Pei J., Pan X., Wei G., Hua Y. (2023). Research progress of glutathione peroxidase family (GPX) in redoxidation. Front. Pharmacol..

[11] Cozza G., Rossetto M., Bosello-Travain V., Maiorino M., Roveri A., Toppo S., Zaccarin M., Zennaro L., Ursini F. (2017). Glutathione peroxidase 4-catalyzed reduction of lipid hydroperoxides in membranes: The polar head of membrane phospholipids binds the enzyme and addresses the fatty acid hydroperoxide group toward the redox center. Free Radic. Biol. Med..

[12] Ursini F., Maiorino M., Valente M., Ferri L., Gregolin C. (1982). Purification from pig liver of a protein which protects liposomes and biomembranes from peroxidative degradation and exhibits glutathione peroxidase activity on phosphatidylcholine hydroperoxides. Biochim. Biophys. Acta (BBA)-Lipids Lipid Metab..

[13] Yang W.S., Stockwell B.R. (2008). Synthetic lethal screening identifies compounds activating iron-dependent, nonapoptotic cell death in oncogenic-RAS-harboring cancer cells. Chem. Biol..

[14] Laurent T.C., Moore E.C., Reichard P. (1964). Enzymatic synthesis of deoxyribonucleotides: IV. Isolation and characterization of thioredoxin, the hydrogen donor from *Escherichia coli* B. J. Biol. Chem..

[15] Moore E.C., Reichard P., Thelander L. (1964). Enzymatic synthesis of deoxyribonucleotides: V. Purification and properties of thioredoxin reductase from *Escherichia coli* B. J. Biol. Chem..

[16] Griendling K.K., Minieri C.A., Ollerenshaw J.D., Alexander R.W. (1994). Angiotensin II stimulates NADH and NADPH oxidase activity in cultured vascular smooth muscle cells. Circ. Res..

[17] Harris J. (1968). Release of a macromolecular protein component from human erythrocyte ghosts. Biochim. Biophys. Acta (BBA)-Biomembr..

[18] Harris J.R. (1980). Torin and cylindrin, two extrinsic proteins of the erythrocyte membrane: A review. Nouv. Rev. Fr. D’Hematologie.

[19] Flohé L., Harris J.R. (2007). Introduction: History of the peroxiredoxins and topical perspectives. Peroxiredoxin Systems: Structures and Functions.

[20] Neumann C.A., Cao J., Manevich Y. (2009). Peroxiredoxin 1 and its role in cell signaling. Cell Cycle.

[21] Fisher A.B. (2018). The phospholipase A^2^. activity of peroxiredoxin 6. J. Lipid Res..

[22] Lee Y.J. (2020). Knockout Mouse Models for Peroxiredoxins. Antioxidant.

[23] McCord J.M., Fridovich I. (1969). Superoxide dismutase: An enzymic function for erythrocuprein (hemocuprein). J. Biol. Chem..

[24] Zelko I.N., Mariani T.J., Folz R.J. (2002). Superoxide dismutase multigene family: A comparison of the CuZn-SOD (SOD1), Mn-SOD (SOD2), and EC-SOD (SOD3) gene structures, evolution, and expression. Free Radic. Biol. Med..

[25] Forstrom J.W., Zakowski J.J., Tappel A.L. (1978). Identification of the catalytic site of rat liver glutathione peroxidase as selenocysteine. Biochemistry.

[26] Zhong L., Holmgren A. (2000). Essential role of selenium in the catalytic activities of mammalian thioredoxin reductase revealed by characterization of recombinant enzymes with selenocysteine mutations. J. Biol. Chem..

[27] Segal A.W., Jones O.T. (1978). Novel cytochrome b system in phagocytic vacuoles of human granulocytes. Nature.

[28] Vermot A., Petit-Härtlein I., Smith S.M., Fieschi F. (2021). NADPH oxidases (NOX): An overview from discovery, molecular mechanisms to physiology and pathology. Antioxidants.

[29] Itoh K., Chiba T., Takahashi S., Ishii T., Igarashi K., Katoh Y., Oyake T., Hayashi N., Satoh K., Hatayama I. (1997). An Nrf2/small Maf heterodimer mediates the induction of phase II detoxifying enzyme genes through antioxidant response elements. Biochem. Biophys. Res. Commun..

[30] Leichert L.I., Gehrke F., Gudiseva H.V., Blackwell T., Ilbert M., Walker A.K., Strahler J.R., Andrews P.C., Jakob U. (2008). Quantifying changes in the thiol redox proteome upon oxidative stress in vivo. Proc. Natl. Acad. Sci. USA.

[31] Rosenwasser S., Graff van Creveld S., Schatz D., Malitsky S., Tzfadia O., Aharoni A., Levin Y., Gabashvili A., Feldmesser E., Vardi A. (2014). Mapping the diatom redox-sensitive proteome provides insight into response to nitrogen stress in the marine environment. Proc. Natl. Acad. Sci. USA.

[32] Jones D.P., Sies H. (2015). The redox code. Antioxid. Redox Signal..

[33] Pulivarthi C.B., Choubey S.S., Pandey S.K., Gautam A.S., Singh R.K. (2023). Receptor tyrosine kinases: An overview. Receptor Tyrosine Kinases in Neurodegenerative and Psychiatric Disorders.

[34] Maiti G.P., Sinha S., Mahmud H., Boysen J., Mendez M.T., Vesely S.K., Holter-Chakrabarty J., Kay N.E., Ghosh A.K. (2021). SIRT3 overexpression and epigenetic silencing of catalase regulate ROS accumulation in CLL cells activating AXL signaling axis. Blood Cancer J..

[35] Kato M., Iwashita T., Takeda K., Akhand A.A., Liu W., Yoshihara M., Asai N., Suzuki H., Takahashi M., Nakashima I. (2000). Ultraviolet Light Induces Redox Reaction–mediated Dimerization and Superactivation of Oncogenic Ret Tyrosine Kinases. Mol. Biol. Cell.

[36] Juarez J.C., Manuia M., Burnett M.E., Betancourt O., Boivin B., Shaw D.E., Tonks N.K., Mazar A.P., Donate F. (2008). Superoxide dismutase 1 (SOD1) is essential for H_2_O_2_-mediated oxidation and inactivation of phosphatases in growth factor signaling. Proc. Natl. Acad. Sci. USA.

[37] World C., Spindel O.N., Berk B.C. (2011). Thioredoxin-interacting protein mediates TRX1 translocation to the plasma membrane in response to tumor necrosis factor-α: A key mechanism for vascular endothelial growth factor receptor-2 transactivation by reactive oxygen species. Arterioscler. Thromb. Vasc. Biol..

[38] Laukkanen M.O., Cammarota F., Esposito T., Salvatore M., Castellone M.D. (2015). Extracellular superoxide dismutase regulates the expression of small gtpase regulatory proteins GEFs, GAPs, and GDI. PLoS ONE.

[39] Lee Y.-J., Cho H.-N., Jeoung D.-I., Soh J.-W., Cho C.K., Bae S., Chung H.-Y., Lee S.-J., Lee Y.-S. (2004). HSP25 overexpression attenuates oxidative stress–induced apoptosis: Roles of ERK1/2 signaling and manganese superoxide dismutase. Free Radic. Biol. Med..

[40] Cardile A., Passarini C., Zanrè V., Fiore A., Menegazzi M. (2023). Hyperforin enhances heme oxygenase-1 expression triggering lipid peroxidation in braf-mutated melanoma cells and hampers the expression of pro-metastatic markers. Antioxidants.

[41] Ma C.-S., Lv Q.-M., Zhang K.-R., Tang Y.-B., Zhang Y.-F., Shen Y., Lei H.-M., Zhu L. (2021). NRF2-GPX4/SOD2 axis imparts resistance to EGFR-tyrosine kinase inhibitors in non-small-cell lung cancer cells. Acta Pharmacol. Sin..

[42] Tran T.-V., Shin E.-J., Nguyen L.T.T., Lee Y., Kim D.-J., Jeong J.H., Jang C.-G., Nah S.-Y., Toriumi K., Nabeshima T. (2018). Protein kinase Cδ gene depletion protects against methamphetamine-induced impairments in recognition memory and ERK1/2 signaling via upregulation of glutathione peroxidase-1 gene. Mol. Neurobiol..

[43] Kang D.H., Lee D.J., Kim J., Lee J.Y., Kim H.-W., Kwon K., Taylor W.R., Jo H., Kang S.W. (2013). Vascular injury involves the overoxidation of peroxiredoxin type II and is recovered by the peroxiredoxin activity mimetic that induces reendothelialization. Circulation.

[44] Heidari E., Tara F., Ghayour-Mobarhan M., Tavalaei S., Boskabadi H., Taghi S.M., Yaghoubi M.A. (2011). The effects of selenium supplementation on serum plasminogen activator inhibitor-1/plasminogen activator inhibitor-2 in pregnant women. Clin. Biochem..

[45] Geraghty P., Hardigan A.A., Wallace A.M., Mirochnitchenko O., Thankachen J., Arellanos L., Thompson V., D’Armiento J.M., Foronjy R.F. (2013). The glutathione peroxidase 1–protein tyrosine phosphatase 1B–protein phosphatase 2A Axis. A key determinant of airway inflammation and alveolar destruction. Am. J. Respir. Cell Mol. Biol..

[46] Duong C., Seow H.J., Bozinovski S., Crack P.J., Anderson G.P., Vlahos R. (2010). Glutathione peroxidase-1 protects against cigarette smoke-induced lung inflammation in mice. Am. J. Physiol. Lung Cell Mol. Physiol..

[47] Yao X.Q., Zhang X.X., Yin Y.Y., Liu B., Luo D.J., Liu D., Chen N.N., Ni Z.F., Wang X., Wang Q. (2011). Glycogen synthase kinase-3β regulates Tyr307 phosphorylation of protein phosphatase-2A via protein tyrosine phosphatase 1B but not Src. Biochem. J..

[48] Wallace A.M., Hardigan A., Geraghty P., Salim S., Gaffney A., Thankachen J., Arellanos L., D’Armiento J.M., Foronjy R.F. (2012). Protein phosphatase 2A regulates innate immune and proteolytic responses to cigarette smoke exposure in the lung. Toxicol. Sci..

[49] Singh K., Maity P., Krug L., Meyer P., Treiber N., Lucas T., Basu A., Kochanek S., Wlaschek M., Geiger H. (2015). Superoxide anion radicals induce IGF-1 resistance through concomitant activation of PTP 1 B and PTEN. EMBO Mol. Med..

[50] Dagnell M., Frijhoff J., Pader I., Augsten M., Boivin B., Xu J., Mandal P.K., Tonks N.K., Hellberg C., Conrad M. (2013). Selective activation of oxidized PTP1B by the thioredoxin system modulates PDGF-β receptor tyrosine kinase signaling. Proc. Natl. Acad. Sci. USA.

[51] Dagnell M., Arnér E.S. (2024). Endogenous electrophiles and peroxymonocarbonate can link tyrosine phosphorylation cascades with the cytosolic TXNRD1 selenoprotein and the KEAP1/NRF2 system. Curr. Opin. Chem. Biol..

[52] Sartelet H., Rougemont A.-L., Fabre M., Castaing M., Duval M., Fetni R., Michiels S., Beaunoyer M., Vassal G. (2011). Activation of the phosphatidylinositol 3′-kinase/AKT pathway in neuroblastoma and its regulation by thioredoxin 1. Hum. Pathol..

[53] Dong A., Wodziak D., Lowe A.W. (2015). Epidermal growth factor receptor (EGFR) signaling requires a specific endoplasmic reticulum thioredoxin for the post-translational control of receptor presentation to the cell surface. J. Biol. Chem..

[54] Laplante M., Sabatini D.M. (2009). mTOR signaling at a glance. J. Cell Sci..

[55] Waterham H.R., Ebberink M.S. (2012). Genetics and molecular basis of human peroxisome biogenesis disorders. Biochim. Biophys. Acta (BBA)-Mol. Basis Dis..

[56] Walker C.L., Pomatto L.C., Tripathi D.N., Davies K.J. (2018). Redox regulation of homeostasis and proteostasis in peroxisomes. Physiol. Rev..

[57] Ye Q., Zhang Y., Cao Y., Wang X., Guo Y., Chen J., Horn J., Ponomareva L.V., Chaiswing L., Shaaban K.A. (2019). Frenolicin B targets peroxiredoxin 1 and glutaredoxin 3 to trigger ROS/4E-BP1-mediated antitumor effects. Cell Chem. Biol..

[58] Dutta R.K., Lee J.N., Maharjan Y., Park C., Choe S.-K., Ho Y.-S., Kwon H.M., Park R. (2022). Catalase-deficient mice induce aging faster through lysosomal dysfunction. Cell Commun. Signal..

[59] Kumar S.L., Kushawaha B., Mohanty A., Kumari A., Kumar A., Beniwal R., Kumar P.K., Athar M., Rao D.K., Rao H.P. (2024). Glutathione peroxidase (GPX1)-Selenocysteine metabolism preserves the follicular fluid’s (FF) redox homeostasis via IGF-1-NMD cascade in follicular ovarian cysts (FOCs). Biochim. Biophys. Acta (BBA)-Mol. Basis Dis..

[60] Agborbesong E., Zhou J.X., Li L.X., Calvet J.P., Li X. (2022). Antioxidant enzyme peroxiredoxin 5 regulates cyst growth and ciliogenesis via modulating Plk1 stability. FASEB J..

[61] Yu Y., Wu T., Lu Y., Zhao W., Zhang J., Chen Q., Ge G., Hua Y., Chen K., Ullah I. (2022). Exosomal thioredoxin-1 from hypoxic human umbilical cord mesenchymal stem cells inhibits ferroptosis in doxorubicin-induced cardiotoxicity via mTORC1 signaling. Free Radic. Biol. Med..

[62] Cho S.Y., Kim S., Son M.-J., Rou W.S., Kim S.H., Eun H.S., Lee B.S. (2019). Clinical significance of the thioredoxin system and thioredoxin-domain-containing protein family in hepatocellular carcinoma. Dig. Dis. Sci..

[63] Wong R.W., Hagen T. (2013). Mechanistic target of rapamycin (mTOR) dependent regulation of thioredoxin interacting protein (TXNIP) transcription in hypoxia. Biochem. Biophys. Res. Commun..

[64] Tsang C.K., Chen M., Cheng X., Qi Y., Chen Y., Das I., Li X., Vallat B., Fu L.-W., Qian C.-N. (2018). SOD1 phosphorylation by mTORC1 couples nutrient sensing and redox regulation. Mol. Cell.

[65] Kong H., Chandel N.S. (2018). To claim growth turf, mTOR says SOD off. Mol. Cell.

[66] Norambuena A., Sun X., Wallrabe H., Cao R., Sun N., Pardo E., Shivange N., Wang D.B., Post L.A., Ferris H.A. (2022). SOD1 mediates lysosome-to-mitochondria communication and its dysregulation by amyloid-β oligomers. Neurobiol. Dis..

[67] König S., Strassheimer F., Brandner N.I., Schröder J.-H., Urban H., Harwart L.F., Hehlgans S., Steinbach J.P., Ronellenfitsch M.W., Luger A.-L. (2024). Superoxide dismutase 1 mediates adaptation to the tumor microenvironment of glioma cells via mammalian target of rapamycin complex 1. Cell Death Discov..

[68] Bandyopadhyay U., Nagy M., Fenton W.A., Horwich A.L. (2014). Absence of lipofuscin in motor neurons of SOD1-linked ALS mice. Proc. Natl. Acad. Sci. USA.

[69] Liu D., Jin X., Yu G., Wang M., Liu L., Zhang W., Wu J., Wang F., Yang J., Luo Q. (2021). Oleanolic acid blocks the purine salvage pathway for cancer therapy by inactivating SOD1 and stimulating lysosomal proteolysis. Mol. Ther.-Oncolytics.

[70] Liu J., Xiao Q., Xiao J., Niu C., Li Y., Zhang X., Zhou Z., Shu G., Yin G. (2022). Wnt/β-catenin signalling: Function, biological mechanisms, and therapeutic opportunities. Signal Transduct. Target. Ther..

[71] Katanaev V.L., Baldin A., Denisenko T.V., Silachev D.N., Ivanova A.E., Sukhikh G.T., Jia L., Ashrafyan L.A. (2023). Cells of the tumor microenvironment speak the Wnt language. Trends Mol. Med..

[72] Larasati Y., Boudou C., Koval A., Katanaev V.L. (2022). Unlocking the Wnt pathway: Therapeutic potential of selective targeting FZD7 in cancer. Drug Discov. Today.

[73] Shaw H.V., Koval A., Katanaev V.L. (2019). Targeting the Wnt signalling pathway in cancer: Prospects and perils. Swiss Med. Wkly..

[74] Kim U., Kim C.Y., Lee J.M., Ryu B., Kim J., Bang J., Ahn N., Park J.H. (2020). Loss of glutathione peroxidase 3 induces ROS and contributes to prostatic hyperplasia in Nkx3. 1 knockout mice. Andrology.

[75] Chang S.N., Lee J.M., Oh H., Park J.H. (2016). Glutathione peroxidase 3 inhibits prostate tumorigenesis in TRAMP mice. Prostate.

[76] Rong X., Zhou Y., Liu Y., Zhao B., Wang B., Wang C., Gong X., Tang P., Lu L., Li Y. (2017). Glutathione peroxidase 4 inhibits Wnt/β-catenin signaling and regulates dorsal organizer formation in zebrafish embryos. Development.

[77] Lin C.-L., Wang J.-Y., Ko J.-Y., Surendran K., Huang Y.-T., Kuo Y.-H., Wang F.-S. (2008). Superoxide destabilization of β-catenin augments apoptosis of high-glucose-stressed mesangial cells. Endocrinology.

[78] Wang F., Fisher S.A., Zhong J., Wu Y., Yang P. (2015). Superoxide dismutase 1 in vivo ameliorates maternal diabetes mellitus–induced apoptosis and heart defects through restoration of impaired Wnt signaling. Circ. Cardiovasc. Genet..

[79] Jung Y.Y., Mohan C.D., Eng H., Narula A.S., Namjoshi O.A., Blough B.E., Rangappa K.S., Sethi G., Kumar A.P., Ahn K.S. (2022). 2, 3, 5, 6-Tetramethylpyrazine targets epithelial-mesenchymal transition by abrogating manganese superoxide dismutase expression and TGFβ-driven signaling cascades in colon cancer cells. Biomolecules.

[80] Wu J., Huang Y., Zhan C., Chen L., Lin Z., Song Z. (2023). Thioredoxin-1 promotes the restoration of alveolar bone in periodontitis with diabetes. iScience.

[81] Funato Y., Michiue T., Asashima M., Miki H. (2006). The thioredoxin-related redox-regulating protein nucleoredoxin inhibits Wnt–β-catenin signalling through dishevelled. Nat. Cell Biol..

[82] Tsuji P.A., Carlson B.A., Yoo M.-H., Naranjo-Suarez S., Xu X.-M., He Y., Asaki E., Seifried H.E., Reinhold W.C., Davis C.D. (2015). The 15kDa selenoprotein and thioredoxin reductase 1 promote colon cancer by different pathways. PLoS ONE.

[83] Ma Y., Li R., Zhang Y., Zhou L., Dai Y. (2014). Knockdown of peroxiredoxin 5 inhibits the growth of osteoarthritic chondrocytes via upregulating Wnt/β-catenin signaling. Free Radic. Biol. Med..

[84] Lu W., Fu Z., Wang H., Feng J., Wei J., Guo J. (2014). Peroxiredoxin 2 knockdown by RNA interference inhibits the growth of colorectal cancer cells by downregulating Wnt/β-catenin signaling. Cancer Lett..

[85] Kang D.H., Lee J.H., Kang S.W. (2017). Survival of APC-mutant colorectal cancer cells requires interaction between tankyrase and a thiol peroxidase, peroxiredoxin II. BMB Rep..

[86] Zheng M.-J., Wang J., Wang H.-M., Gao L.-L., Li X., Zhang W.-C., Gou R., Guo Q., Nie X., Liu J.-J. (2018). Decreased expression of peroxiredoxin1 inhibits proliferation, invasion, and metastasis of ovarian cancer cell. OncoTargets Ther..

[87] Liu Y., Kwon T., Kim J.-S., Chandimali N., Jin Y.-H., Gong Y.-X., Xie D.-P., Han Y.-H., Jin M.-H., Shen G.-N. (2019). Peroxiredoxin V Reduces β-Lapachone-induced apoptosis of colon cancer cells. Anticancer Res..

[88] Xu J., Su Q., Gao M., Liang Q., Li J., Chen X. (2019). Differential expression and effects of peroxiredoxin-6 on drug resistance and cancer stem cell-like properties in non-small cell lung cancer. OncoTargets Ther..

[89] Lee T.H., Jin J.-O., Yu K.J., Kim H.S., Lee P.C.-W. (2019). Inhibition of peroxiredoxin 2 suppresses Wnt/β-catenin signaling in gastric cancer. Biochem. Biophys. Res. Commun..

[90] Chandimali N., Huynh D.L., Zhang J.J., Lee J.C., Yu D.-Y., Jeong D.K., Kwon T. (2019). MicroRNA-122 negatively associates with peroxiredoxin-II expression in human gefitinib-resistant lung cancer stem cells. Cancer Gene Ther..

[91] Peng L., Xiong Y., Wang R., Xiang L., Zhou H., Fu Z. (2021). The critical role of peroxiredoxin-2 in colon cancer stem cells. Aging.

[92] Yang X., Xiang X., Xu G., Zhou S., An T., Huang Z. (2023). Silencing of peroxiredoxin 2 suppresses proliferation and Wnt/β-catenin pathway, and induces senescence in hepatocellular carcinoma. Oncol. Res..

[93] Kipp A., Banning A., Brigelius-Flohé R. (2007). Activation of the glutathione peroxidase 2 (GPx2) promoter by β-catenin. Biol. Chem..

[94] Giera S., Braeuning A., Köhle C., Bursch W., Metzger U., Buchmann A., Schwarz M. (2010). Wnt/β-catenin signaling activates and determines hepatic zonal expression of glutathione S-transferases in mouse liver. Toxicol. Sci..

[95] Braeuning A. (2012). Interplay of β-catenin with xenobiotic-sensing receptors and its role in glutathione S-transferase expression. Curr. Drug Metab..

[96] Balogun O., Shao D., Carson M., King T., Kosar K., Zhang R., Zeng G., Cornuet P., Goel C., Lee E. (2024). Loss of β-catenin reveals a role for glutathione in regulating oxidative stress during cholestatic liver disease. Hepatol. Commun..

[97] Schmierer B., Hill C.S. (2007). TGFβ–SMAD signal transduction: Molecular specificity and functional flexibility. Nat. Rev. Mol. Cell Biol..

[98] Zhang J., Bai J., Zhou Q., Hu Y., Wang Q., Yang L., Chen H., An H., Zhou C., Wang Y. (2022). Glutathione prevents high glucose-induced pancreatic fibrosis by suppressing pancreatic stellate cell activation via the ROS/TGFβ/SMAD pathway. Cell Death Dis..

[99] Chang C., Cheng Y.-Y., Kamlapurkar S., White S., Tang P.W., Elhaw A.T., Javed Z., Aird K.M., Mythreye K., Phaëton R. (2024). GPX3 supports ovarian cancer tumor progression in vivo and promotes expression of GDF15. Gynecol. Oncol..

[100] Ye Z., Cheng M., Lian W., Leng Y., Qin X., Wang Y., Zhou P., Liu X., Peng T., Wang R. (2025). GPX4 deficiency-induced ferroptosis drives endometrial epithelial fibrosis in polycystic ovary syndrome. Redox Biol..

[101] Buday A., Orsy P., Godó M., Mózes M., Kökény G., Lacza Z., Koller Á., Ungvári Z., Gross M.-L., Benyó Z. (2010). Elevated systemic TGF-β impairs aortic vasomotor function through activation of NADPH oxidase-driven superoxide production and leads to hypertension, myocardial remodeling, and increased plaque formation in apoE^−^/^−^ mice. Am. J. Physiol. Heart Circ. Physiol..

[102] Andrade B.B., Araújo-Santos T., Luz N.F., Khouri R., Bozza M.T., Camargo L., Barral A., Borges V.M., Barral-Netto M. (2010). Heme impairs prostaglandin E2 and TGF-β production by human mononuclear cells via Cu/Zn superoxide dismutase: Insight into the pathogenesis of severe malaria. J. Immunol..

[103] Fatma N., Kubo E., Sharma P., Beier D., Singh D. (2005). Impaired homeostasis and phenotypic abnormalities in Prdx6^−^/^−^ mice lens epithelial cells by reactive oxygen species: Increased expression and activation of TGFβ. Cell Death Differ..

[104] Sun X., Mu Q., Yang F., Liu M., Zhou B. (2024). The effects of thioredoxin peroxidase from Cysticercus cellulosae excretory-secretory antigens on TGF-β signaling pathway and Th17 cells differentiation in Jurkat cells by transcriptomics. Parasitol. Res..

[105] Choi H.-I., Ma S.K., Bae E.H., Lee J., Kim S.W. (2016). Peroxiredoxin 5 protects TGF-β induced fibrosis by inhibiting Stat3 activation in rat kidney interstitial fibroblast cells. PLoS ONE.

[106] Choi H.-I., Kim D.-H., Park J.S., Kim I.J., Kim C.S., Bae E.H., Ma S.K., Lee T.-H., Kim S.W. (2019). Peroxiredoxin V (PrdxV) negatively regulates EGFR/Stat3-mediated fibrogenesis via a Cys48-dependent interaction between PrdxV and Stat3. Sci. Rep..

[107] Manoharan R., Seong H.-A., Ha H. (2013). Thioredoxin inhibits MPK38-induced ASK1, TGF-β, and p53 function in a phosphorylation-dependent manner. Free Radic. Biol. Med..

[108] Liu Q., Sun Y., Zhu Y., Qiao S., Cai J., Zhang Z. (2022). Melatonin relieves liver fibrosis induced by Txnrd3 knockdown and nickel exposure via IRE1/NF-kB/NLRP3 and PERK/TGF-β1 axis activation. Life Sci..

[109] Ishikawa F., Kaneko E., Sugimoto T., Ishijima T., Wakamatsu M., Yuasa A., Sampei R., Mori K., Nose K., Shibanuma M. (2014). A mitochondrial thioredoxin-sensitive mechanism regulates TGF-β-mediated gene expression associated with epithelial–mesenchymal transition. Biochem. Biophys. Res. Commun..

[110] Liu T., Zhang L., Joo D., Sun S.-C. (2017). NF-κB signaling in inflammation. Signal Transduct. Target. Ther..

[111] Schreck R., Rieber P., Baeuerle P.A. (1991). Reactive oxygen intermediates as apparently widely used messengers in the activation of the NF-kappa B transcription factor and HIV-1. EMBO J..

[112] Anderson M.T., Staal F., Gitler C., Herzenberg L.A., Herzenberg L.A. (1994). Separation of oxidant-initiated and redox-regulated steps in the NF-kappa B signal transduction pathway. Proc. Natl. Acad. Sci. USA.

[113] Wang X., Tao Y., Huang Y., Zhan K., Xue M., Wang Y., Ruan D., Liang Y., Huang X., Lin J. (2017). Catalase ameliorates diabetes-induced cardiac injury through reduced p65/RelA-mediated transcription of BECN1. J. Cell. Mol. Med..

[114] Cong W., Ruan D., Xuan Y., Niu C., Tao Y., Wang Y., Zhan K., Cai L., Jin L., Tan Y. (2015). Cardiac-specific overexpression of catalase prevents diabetes-induced pathological changes by inhibiting NF-κB signaling activation in the heart. J. Mol. Cell. Cardiol..

[115] Lüpertz R., Chovolou Y., Kampkötter A., Wätjen W., Kahl R. (2008). Catalase overexpression impairs TNF-α induced NF-κB activation and sensitizes MCF-7 cells against TNF-α. J. Cell. Biochem..

[116] Moon S.W., Ahn C.-B., Oh Y., Je J.-Y. (2019). Lotus (*Nelumbo nucifera*) seed protein isolate exerts anti-inflammatory and antioxidant effects in LPS-stimulated RAW264. 7 macrophages via inhibiting NF-κB and MAPK pathways, and upregulating catalase activity. Int. J. Biol. Macromol..

[117] Imran M., Arshad M.S., Butt M.S., Kwon J.-H., Arshad M.U., Sultan M.T. (2017). Mangiferin: A natural miracle bioactive compound against lifestyle related disorders. Lipids Health Dis..

[118] Sahoo B.K., Zaidi A.H., Gupta P., Mokhamatam R.B., Raviprakash N., Mahali S.K., Manna S.K. (2015). A natural xanthone increases catalase activity but decreases NF-kappa B and lipid peroxidation in U-937 and HepG2 cell lines. Eur. J. Pharmacol..

[119] Shah M.H., Liu G.-S., Thompson E.W., Dusting G.J., Peshavariya H.M. (2015). Differential effects of superoxide dismutase and superoxide dismutase/catalase mimetics on human breast cancer cells. Breast Cancer Res. Treat..

[120] Shi X., Shi Z., Huang H., Zhu H., Zhou P., Zhu H., Ju D. (2014). Ability of recombinant human catalase to suppress inflammation of the murine lung induced by influenza A. Inflammation.

[121] Zuo X., Guo X., Gu Y., Zhao D., Zou Z., Shen Y., He C., Rong Y., Xu C., Wang F. (2024). A novel catalase nanogels for effective treatment of neutrophilic asthma. Adv. Funct. Mater..

[122] Senger N., C Parletta A., Marques B.V., Akamine E.H., Diniz G.P., Campagnole-Santos M.J., Santos R.A., Barreto-Chaves M.L.M. (2021). Angiotensin-(1-7) prevents T3-induced cardiomyocyte hypertrophy by upregulating FOXO3/SOD1/catalase and downregulating NF-ĸB. J. Cell. Physiol..

[123] Han W., Fessel J.P., Sherrill T., Kocurek E.G., Yull F.E., Blackwell T.S. (2020). Enhanced expression of catalase in mitochondria modulates NF-κB–Dependent lung inflammation through alteration of metabolic activity in macrophages. J. Immunol..

[124] Mu Y., Maharjan Y., Kumar Dutta R., Wei X., Kim J.H., Son J., Park C., Park R. (2021). Pharmacological inhibition of catalase induces peroxisome leakage and suppression of LPS induced inflammatory response in Raw 264.7 cell. PLoS ONE.

[125] Jang B.-C., Paik J.-H., Kim S.-P., Shin D.-H., Song D.-K., Park J.-G., Suh M.-H., Park J.-W., Suh S.-I. (2005). Catalase induced expression of inflammatory mediators via activation of NF-κB, PI3K/AKT, p70S6K, and JNKs in BV2 microglia. Cell. Signal..

[126] Jang B.-C., Paik J.-H., Kim S.-P., Bae J.-H., Mun K.-C., Song D.-K., Cho C.-H., Shin D.-H., Kwon T.K., Park J.-W. (2004). Catalase induces the expression of inducible nitric oxide synthase through activation of NF-κB and PI3K signaling pathway in Raw 264.7 cells. Biochem. Pharmacol..

[127] Jang B.-C., Kim D.-H., Park J.-W., Kwon T.K., Kim S.-P., Song D.-K., Park J.-G., Bae J.-H., Mun K.-C., Baek W.-K. (2004). Induction of cyclooxygenase-2 in macrophages by catalase: Role of NF-κB and PI3K signaling pathways. Biochem. Biophys. Res. Commun..

[128] Crack P.J., Taylor J.M., Ali U., Mansell A., Hertzog P.J. (2006). Potential contribution of NF-κB in neuronal cell death in the glutathione peroxidase-1 knockout mouse in response to ischemia-reperfusion injury. Stroke.

[129] Brigelius-Flohé R. (2006). Glutathione peroxidases and redox-regulated transcription factors. Biol. Chem..

[130] Gong G., Meplan C., Gautrey H., Hall J., Hesketh J. (2012). Differential effects of selenium and knock-down of glutathione peroxidases on TNFα and flagellin inflammatory responses in gut epithelial cells. Genes Nutr..

[131] Peng D.-F., Hu T.-L., Soutto M., Belkhiri A., El-Rifai W. (2014). Glutathione peroxidase 7 suppresses bile salt-induced expression of pro-inflammatory cytokines in Barrett’s carcinogenesis. J. Cancer.

[132] Peng D.-F., Hu T.-L., Soutto M., Belkhiri A., El-Rifai W. (2014). Loss of glutathione peroxidase 7 promotes TNF-α-induced NF-κB activation in Barrett’s carcinogenesis. Carcinogenesis.

[133] Li C., Deng X., Xie X., Liu Y., Friedmann Angeli J.P., Lai L. (2018). Activation of glutathione peroxidase 4 as a novel anti-inflammatory strategy. Front. Pharmacol..

[134] Sharma N., Shin E.-J., Pham D.T., Sharma G., Dang D.-K., Duong C.X., Kang S.W., Nah S.-Y., Jang C.-G., Lei X.G. (2021). GPx-1-encoded adenoviral vector attenuates dopaminergic impairments induced by methamphetamine in GPx-1 knockout mice through modulation of NF-κB transcription factor. Food Chem. Toxicol..

[135] Wang X., Han Y., Chen F., Wang M., Xiao Y., Wang H., Xu L., Liu W. (2022). Glutathione peroxidase 1 protects against peroxynitrite-induced spiral ganglion neuron damage through attenuating NF-κB pathway activation. Front. Cell. Neurosci..

[136] Zhang J., Peng Z., Cheng D., Yao W., Li H., Zhang Q., Guo R., Li K., Zou L., Wang J.-S. (2024). Lepidium meyenii (Maca) polysaccharides mitigate liver toxicity of aflatoxin B1 through activation of NRF-2/GPX and AhR/STAT3 signaling pathways. Toxicon.

[137] Yang H., Wang Z., Li L., Wang X., Wei X., Gou S., Ding Z., Cai Z., Ling Q., Hoffmann P.R. (2024). Mannose coated selenium nanoparticles normalize intestinal homeostasis in mice and mitigate colitis by inhibiting NF-κB activation and enhancing glutathione peroxidase expression. J. Nanobiotechnology.

[138] Izuhara K., Suzuki S., Ogawa M., Nunomura S., Nanri Y., Mitamura Y., Yoshihara T. (2017). The significance of hypothiocyanite production via the pendrin/DUOX/peroxidase pathway in the pathogenesis of asthma. Oxidative Med. Cell. Longev..

[139] Wang J.-G., Mahmud S.A., Nguyen J., Slungaard A. (2006). Thiocyanate-dependent induction of endothelial cell adhesion molecule expression by phagocyte peroxidases: A novel HOSCN-specific oxidant mechanism to amplify inflammation. J. Immunol..

[140] Wang J.-G., Mahmud S.A., Thompson J.A., Geng J.-G., Key N.S., Slungaard A. (2006). The principal eosinophil peroxidase product, HOSCN, is a uniquely potent phagocyte oxidant inducer of endothelial cell tissue factor activity: A potential mechanism for thrombosis in eosinophilic inflammatory states. Blood.

[141] Akasheh N., Walsh M.-T., Costello R.W. (2014). Eosinophil peroxidase induces expression of cholinergic genes via cell surface neural interactions. Mol. Immunol..

[142] Jin D.-Y., Chae H.Z., Rhee S.G., Jeang K.-T. (1997). Regulatory role for a novel human thioredoxin peroxidase in NF-κB activation. J. Biol. Chem..

[143] Kim Y., Kim B.H., Lee H., Jeon B., Lee Y.S., Kwon M.-J., Kim T.-Y. (2011). Regulation of skin inflammation and angiogenesis by EC-SOD via HIF-1α and NF-κB pathways. Free Radic. Biol. Med..

[144] Li W., Cao L., Han L., Xu Q., Ma Q. (2015). Superoxide dismutase promotes the epithelial-mesenchymal transition of pancreatic cancer cells via activation of the H2O2/ERK/NF-κB axis. Int. J. Oncol..

[145] Abu-Zeid E.H., El-Hady E.W., Ahmed G.A., Abd-Elhakim Y.M., Ibrahim D., Abd-Allah N.A., Arisha A.H., Sobh M.S., Abo-Elmaaty A.M. (2024). Nicotine exacerbates liver damage in a mice model of Ehrlich ascites carcinoma through shifting SOD/NF-κB/caspase-3 pathways: Ameliorating role of Chlorella vulgaris. Naunyn-Schmiedeberg’s Arch. Pharmacol..

[146] Heilman J.M., Burke T.J., McClain C.J., Watson W.H. (2011). Transactivation of gene expression by NF-κB is dependent on thioredoxin reductase activity. Free Radic. Biol. Med..

[147] Sakurai A., Yuasa K., Shoji Y., Himeno S., Tsujimoto M., Kunimoto M., Imura N., Hara S. (2004). Overexpression of thioredoxin reductase 1 regulates NF-κB activation. J. Cell. Physiol..

[148] Hansen J.M., Zhang H., Jones D.P. (2006). Mitochondrial thioredoxin-2 has a key role in determining tumor necrosis factor-α–induced reactive oxygen species generation, NF-κB activation, and apoptosis. Toxicol. Sci..

[149] Wang X., Xing Y., Tang Z., Tang Y., Shen J., Zhang F. (2020). Thioredoxin-2 impacts the inflammatory response via suppression of NF-κB and MAPK signaling in sepsis shock. Biochem. Biophys. Res. Commun..

[150] Hao X., Liu M., Zhang X., Yu H., Fang Z., Gao X., Chen M., Shao Q., Gao W., Lei L. (2024). Thioredoxin-2 suppresses hydrogen peroxide–activated nuclear factor kappa B signaling via alleviating oxidative stress in bovine adipocytes. J. Dairy. Sci..

[151] Kelleher Z.T., Sha Y., Foster M.W., Foster W.M., Forrester M.T., Marshall H.E. (2014). Thioredoxin-mediated denitrosylation regulates cytokine-induced nuclear factor κB (NF-κB) activation. J. Biol. Chem..

[152] Lu Y., Zhao X., Luo G., Shen G., Li K., Ren G., Pan Y., Wang X., Fan D. (2015). Thioredoxin-like protein 2b facilitates colon cancer cell proliferation and inhibits apoptosis via NF-κB pathway. Cancer Lett..

[153] Zhu Z., Chen X., Sun J., Li Q., Lian X., Li S., Wang Y., Tian L. (2019). Inhibition of nuclear thioredoxin aggregation attenuates PM2. 5-induced NF-κB activation and pro-inflammatory responses. Free Radic. Biol. Med..

[154] Yang H., Li L., Jiao Y., Zhang Y., Wang Y., Zhu K., Sun C. (2021). Thioredoxin-1 mediates neuroprotection of Schisanhenol against MPP+-induced apoptosis via suppression of ASK1-P38-NF-κB pathway in SH-SY5Y cells. Sci. Rep..

[155] Xiao Z., Xu Q., Wang H., Zhou X., Zhu Y., Bao C., Chen L., Zhang P., Lin M., Ji C. (2023). Thioredoxin domain-containing protein 9 protects cells against UV-B-provoked apoptosis via NF-κB/p65 activation in cutaneous squamous cell carcinoma. Oncol. Res..

[156] Tan W.-H., Pyeritz R.E., Korf B.R., Grody W.W. (2025). CHAPTER 1—Human Developmental Genetics. Emery and Rimoin’s Principles and Practice of Medical Genetics and Genomics.

[157] Wang R., Wei J., Zhang S., Wu X., Guo J., Liu M., Du K., Xu J., Peng L., Lv Z. (2016). Peroxiredoxin 2 is essential for maintaining cancer stem cell-like phenotype through activation of Hedgehog signaling pathway in colon cancer. Oncotarget.

[158] Lee D.H., Jung Y.Y., Park M.H., Jo M.R., Han S.B., Yoon D.Y., Roh Y.S., Hong J.T. (2019). Peroxiredoxin 6 confers protection against nonalcoholic fatty liver disease through maintaining mitochondrial function. Antioxid. Redox Signal..

[159] Young B., Purcell C., Kuang Y.-Q., Charette N., Dupré D.J. (2015). Superoxide dismutase 1 regulation of CXCR4-mediated signaling in prostate cancer cells is dependent on cellular oxidative state. Cell. Physiol. Biochem..

[160] Schattauer S.S., Land B.B., Reichard K.L., Abraham A.D., Burgeno L.M., Kuhar J.R., Phillips P.E., Ong S.E., Chavkin C. (2017). Peroxiredoxin 6 mediates Gαi protein-coupled receptor inactivation by cJun kinase. Nat. Commun..

