# Supplementary material for: Beyond Antioxidants: How Redox Pathways Shape Cellular Signaling and Disease Outcomes
Source: Antioxidants (Basel). 2025 Sep 22;14(9):1142. doi: 10.3390/antiox14091142 (PMC12466437; doi:10.3390/antiox14091142)
Supplement: Supplementary file 1 [file antioxidants-14-01142-s001.zip › antioxidants-3846912-supplementary.pdf]

NCT Number	Enzyme	Drug / Intervention	Status	Indication	Phase	Country	Year (Completion/ Estimate)	Link
NCT06618209	SOD	DIAGNOSTIC_TEST: physical and chemical methods, biochemical methods, Oral Findings, Pediatric Oral Health Quality of Life	Completed	Primary Ciliary Dyskinesia / Caries,Dental / Quality of Life / Saliva Altered / Erosion, Dental Enamel	Observational [Patient Registry]	Turkey	2024	https://clinicaltrials.gov/study/NCT06618209
NCT06329284	SOD	DIAGNOSTIC_TEST: ELISA	Completed	Pre-Eclampsia	Not Applicable	Egypt	2024	https://clinicaltrials.gov/study/NCT06329284
NCT06324071	SOD	DIETARY_SUPPLEMENT: Sham, Marine microalgae Tetraselmis chuii with high Superoxide Dismutase (SOD) activity	Recruiting	Male Infertility	Not Applicable	Spain	2025	https://clinicaltrials.gov/study/NCT06324071
NCT06131918	SOD	DRUG: Resveratrol, Alpha lipoic acid, Super oxide dismutase, Resveratrol, Alpha lipoic acid, Super Oxide Dismutase	Completed	Peripheral Diabetic Neuropathy	2	Pakistan	2024	https://clinicaltrials.gov/study/NCT06131918
NCT05984771	SOD	DIETARY_SUPPLEMENT: Epineuron|OTHER: Placebo group	Completed	Diabetic Neuropathies / Diabetes Mellitus / Dietary Supplement	Not Applicable	Greece	2022	https://clinicaltrials.gov/study/NCT05984771
NCT05711810	SOD	DRUG: Nifedipine; enalapril maleate; lansoprazole; metoprolol succinate; duloxetine hydrochloride; superoxide dismutase. DIETARY_SUPPLEMENT: Coenzyme Q10; d-alpha-tocopherol acetate; omega-3. BEHAVIORAL: low mobility. diagnostic_test: Kangzhu BPCB0A-3A.	Completed	Severe Acute Respiratory Syndrome-related Coronavirus / Renal Dialysis / Vaccines / Myocarditis Allergic / Infection Viral	4	China	2023	https://clinicaltrials.gov/study/NCT05711810
NCT05575739	SOD	DIAGNOSTIC_TEST: Measurement of oxidative stress levels (Malondialdehide, glutathione disulphate, total antioxidant capacity, superoxide dismutase and carbonylated proteins)	Unkown status	IVF / Pregnancy / Oxidative Stress / Fertility Disorders	Observational	Romania	2023	https://clinicaltrials.gov/study/NCT05575739
NCT05558878	SOD	DRUG: Ambroxol Oral Product	Unkown status	Diabetic Neuropathy Peripheral	Not Applicable	Egypt	2023	https://clinicaltrials.gov/study/NCT05558878
NCT05357157	SOD	OTHER: Electroacupuncture|DIETARY_SUPPLEMENT: Nutrition	Recruiting	Fibromyalgia / Electroacupuncture / Acupuncture / Hyperalgesia	Observational	Greece	2026	https://clinicaltrials.gov/study/NCT05357157
NCT05215444	SOD	DIAGNOSTIC_TEST: some prognostic factors	Completed	Aluminum Phosphide Poisoning	Observational	Egypt	2021	https://clinicaltrials.gov/study/NCT05215444
NCT05208879	SOD	BIOLOGICAL: Characterization of carotenoid status and plasma levels of oxidative stress markers in patients (case group only)|OTHER: Characterization, evaluation and comparison of macular pigment density	Recruiting	Primary Intestinal Hypocholesterolemia / Abetalipoproteinemia / Chylomicron Retention Disease	Observational	France	2026	https://clinicaltrials.gov/study/NCT05208879
NCT05205005	SOD	DRUG: Endocalyx, Placebo	Completed	Diabetes Mellitus, Type 2	2 and 3	United States	2024	https://clinicaltrials.gov/study/NCT05205005
NCT05178953	SOD	DRUG: Zinc Sulfate, Pentoxifylline	Unkown status	Men Infertility	Early Phase 1	Iran	2022	https://clinicaltrials.gov/study/NCT05178953
NCT05060120	SOD	DIAGNOSTIC_TEST: biomakers for ovarian torsion, peritoneal fluid proteins analysis	Unkown status	Ovary Torsion / Adnexal Torsion	Observational	Israel	2024	https://clinicaltrials.gov/study/NCT05060120
NCT04919850	SOD	DIETARY_SUPPLEMENT: Saccharomyces boulardii and SOD|COMBINATION_PRODUCT: Placebo	Completed	Obesity / Superoxide Dismutase	Not Applicable	Italy	2020	https://clinicaltrials.gov/study/NCT04919850
NCT04864314	SOD	DIETARY_SUPPLEMENT: Marine microalgae Tetraselmis chuii with high Superoxide Dismutase (SOD) activity, Sham	Completed	Infertility, Male	Not Applicable	Spain	2024	https://clinicaltrials.gov/study/NCT04864314

NCT04856982	SOD	DRUG: Tofersen, Placebo	Active, not recruiting	Amyotrophic Lateral Sclerosis Associated With a SOD1 Gene Mutation	3	United States	2027	https://clinicaltrials.gov/study/NCT04856982
NCT04748887	SOD	OTHER: oxidative stess markers	Completed	Role of Oxidative Stress in COPD	Observational [Patient Registry]	Egypt	2019	https://clinicaltrials.gov/study/NCT04748887
NCT04669119	SOD	DIETARY_SUPPLEMENT: Bromelain/Boswellia Serrata Casperome, Centella Asiatica/Vitamins, Placebo	Unkown status	Breast Cancer Female / Pain, Postoperative	4	Italy	2022	https://clinicaltrials.gov/study/NCT04669119
NCT04568161	SOD	PROCEDURE: Physical Characteristics, Muscular Sympathetic Nervous Activity|DIAGNOSTIC_TEST: Cardiac Function, Heart rate, Blood pressure, Blood Assessments, Muscle blood flow, Endothelium-dependent vascular function, Vascular intima-media thickness, Physical Capacity|DRUG: Anthracycline & Cyclophosphamide treatment scheme	Completed	Cardiotoxicity / Cardiovascular Disease / Neurovascular Disorder / Endothelial Disfunction / Breast Cancer	Not Applicable	Brazil	2025	https://clinicaltrials.gov/study/NCT04568161
NCT04466657	SOD	DIETARY_SUPPLEMENT: Antioxidation Therapy|OTHER: Standard of Care	Withdrawn	Covid-19	Not Applicable	Nigeria	2021	https://clinicaltrials.gov/study/NCT04466657
NCT04314596	SOD	OTHER: Cross Training Course|DIETARY_SUPPLEMENT: Oceanix	Completed	Oxidative Stress / Muscle Strength / Resistance Training / Body Composition / Muscle Damage	Not Applicable	United States	2020	https://clinicaltrials.gov/study/NCT04314596
NCT04315077	SOD	DIETARY_SUPPLEMENT: Oceanix|OTHER: Resistance Training	Completed	Oxidative Stress / Muscle Strength / Resistance Training / Muscle Damage / Immune Suppression	Not Applicable	United States	2019	https://clinicaltrials.gov/study/NCT04315077
NCT04180332	SOD, Catalase	DIAGNOSTIC_TEST: Blood sample	Completed	Periodontal Diseases / Diabetes Mellitus / Enzyme Disorder	Not Applicable	India	2018	https://clinicaltrials.gov/study/NCT04180332
NCT04136821	SOD	DIETARY_SUPPLEMENT: Oceanix|OTHER: Resistance Training	Completed	Oxidative Stress / Muscle Strength / Resistance Training / Body Composition / Muscle Damage	Not Applicable	United States	2020	https://clinicaltrials.gov/study/NCT04136821
NCT03995732	SOD	DRUG: PC-SOD, placebo	Unkown status	Myocardial Reperfusion Injury	2	China	2021	https://clinicaltrials.gov/study/NCT03995732
NCT03941808	SOD	DRUG: Glisodin tablet, Placebo tablet	Completed	Vitiligo	Not Applicable	France	2020	https://clinicaltrials.gov/study/NCT03941808
NCT03878433	SOD	DRUG: Glisodin|OTHER: placebo	Completed	Melasma	Not Applicable	France	2020	https://clinicaltrials.gov/study/NCT03878433
NCT03815305	SOD	DRUG: Centella Asiatica Extract, Topical CA, Petroleum jelly	Unkown status	Xerosis Cutis / Diabetes Mellitus, Type 2	4	Indonesia	2019	https://clinicaltrials.gov/study/NCT03815305
NCT03762031	SOD	DRUG: Placebo, GC4711	Completed	Healthy	1	Austrailia	2020	https://clinicaltrials.gov/study/NCT03762031
NCT03689712	SOD	DRUG: GC4419, Placebo	Completed	Oral Mucositis	3	United States	2023	https://clinicaltrials.gov/study/NCT03689712
NCT03522532	SOD	PROCEDURE: Amalgam placement, Tetric EvoCeram placement, Beautifil placement, Zinc phosphate cement placement, Zinc polycarboxylate cement placement, Glass ionomer cement placement	Completed	Pharmacological Action	Not Applicable	Serbia	2017	https://clinicaltrials.gov/study/NCT03522532

NCT03505411	GPx, SOD	DIETARY_SUPPLEMENT: melatonin supplementation	Completed	Antioxidants / Oxidative Stress / Inflammation / Lipid Peroxidation	Not Applicable	No location data	2012	https://clinicaltrials.gov/study/NCT03505411
NCT03457974	SOD	OTHER: saliva	Completed	Cardiac Disease / Dental Caries	Not Applicable	No location data	2012	https://clinicaltrials.gov/study/NCT03457974
NCT03194139	SOD	DRUG: GC4711, GC4419	Completed	Healthy Volunteers / Healthy	1	Austrailia	2017	https://clinicaltrials.gov/study/NCT03194139
NCT03164109	SOD	DRUG: GC4419 IV|DRUG: Placebo|DRUG: Oral moxifloxacin	Completed	Healthy Volunteers / Healthy	1	Austrailia	2017	https://clinicaltrials.gov/study/NCT03164109
NCT03163173	SOD	DRUG: GC4419	Completed	Healthy Volunteers / Healthy	1	United States	2017	https://clinicaltrials.gov/study/NCT03163173
NCT03155438	GPx, SOD	DIAGNOSTIC_TEST: Oxidative stress-related gene expression (RNA expression), oocyte competence biomarkers	Unkown status	Low Responder	Observational	Thailand	2018	https://clinicaltrials.gov/study/NCT03155438
NCT03099824	SOD	DRUG: GC4711 oral capsule G-101; GC4711 oral capsule G-111; GC4711 oral capsule G-112; GC4711 oral capsule G-119; GC4711 oral capsule G-125; IV GC4419; IV GC4711.	Completed	Healthy Volunteers	1	Austrailia	2020	https://clinicaltrials.gov/study/NCT03099824
NCT03096756	SOD	DRUG: GC4702 dry powder; GC4702 lipid suspension; GC4702 lipid suspension - Part 2. OTHER: GC4419 IV; placebo dry powder; placebo lipid suspension; fed condition; fasting condition.	Completed	Healthy Volunteer	1	Austrailia	2016	https://clinicaltrials.gov/study/NCT03096756
NCT03071302	SOD	OTHER: EVALUATION OF VSX1, SOD1, and TIMP3 genes	Completed	Keratoconus / Keratoconus, Unspecified, Bilateral	Not Applicable	Brazil	2016	https://clinicaltrials.gov/study/NCT03071302
NCT03070119	SOD	DRUG: Tofersen	Completed	ALS Caused by Superoxide Dismutase 1 (SOD1) Mutation	3	United States	2024	https://clinicaltrials.gov/study/NCT03070119
NCT02880657	SOD	DIETARY_SUPPLEMENT: SODB, Placebo	Unkown status	Healthy Volunteers	Not Applicable	No location data	2018	https://clinicaltrials.gov/study/NCT02880657
NCT02753582	SOD	DIETARY_SUPPLEMENT: SOD+Gliadin Capsule, Placebo	Unkown status	Pre-frail Elderly	4	Indonesia	2017	https://clinicaltrials.gov/study/NCT02753582
NCT02667691	SOD	DIETARY_SUPPLEMENT: SODB Dimpless, OTHER: caloric restriction	Unkown status	Overweight	Not Applicable	No location data	2018	https://clinicaltrials.gov/study/NCT02667691
NCT02623699	SOD	DRUG: Toferse, Placebo	Completed	Amyotrophic Lateral Sclerosis	3	United States	2021	https://clinicaltrials.gov/study/NCT02623699
NCT02508389	SOD	DRUG: low-dose GC4419 30 mg/day; high-dose GC4419 90 mg/day; placebo; cisplatin. radiation: intensity-modulated radiation therapy	Completed	Radiation Induced Oral Mucositis	2	United States	2019	https://clinicaltrials.gov/study/NCT02508389
NCT02442453	SOD	DRUG: Topical application of curenext gel, Placebo	Completed	Chronic Periodontitis	4	No location data	2014	https://clinicaltrials.gov/study/NCT02442453
NCT02149758	SOD	DRUG: Etoricoxib	Completed	Cyclooxygenase Two Inhibitors / Etoricoxib / Periodontitis / Superoxide Dismutase / Saliva	4	India	2013	https://clinicaltrials.gov/study/NCT02149758
NCT02066337	SOD	PROCEDURE: scaling and root planing|DRUG: placebo gel, Ozonated olive oil gel	Completed	Periodontal Diseases	2	Egypt	2014	https://clinicaltrials.gov/study/NCT02066337
NCT01921426	SOD	DRUG: GC4419	Completed	Squamous Cell Carcinoma of the Oral Cavity / Squamous	1	United States	2016	https://clinicaltrials.gov/study/NCT01921426

				Cell Carcinoma of the Oropharynx				
NCT01771991	SOD	DRUG: Topical Sodermix Dismutase in the form of Sodermix (SOD), Placebo	Completed	Radiation Induced Fibrosis to the Head and Neck	Not Applicable	United States	2013	https://clinicaltrials.gov/study/NCT01771991
NCT01513278	SOD	DRUG: APN201, Placebo	Completed	Radiation Induced Dermatitis	1 and 2	Austria	2012	https://clinicaltrials.gov/study/NCT01513278
NCT01042262	SOD	OTHER: Oxygen, Placebo	Completed	Oxidative Stress / Fetal Distress	Not Applicable	United States	2009	https://clinicaltrials.gov/study/NCT01042262
NCT01041222	SOD	DRUG: ISIS 333611	Completed	Familial Amyotrophic Lateral Sclerosis	1	United States	2012	https://clinicaltrials.gov/study/NCT01041222
NCT00898274	GPx, SOD	OTHER: immunoenzyme technique, laboratory biomarker analysis	Completed	Precancerous Condition / Prostate Cancer	Observational	United States	2011	https://clinicaltrials.gov/study/NCT00898274
NCT00860743	SOD	DRUG: Antioxidant cocktail	Completed	Sleep Apnea Syndromes	Early Phase 1	United States	2013	https://clinicaltrials.gov/study/NCT00860743
NCT00800995	SOD	DIETARY_SUPPLEMENT: SOD, Sham	Completed	Age Related Macular Degeneration	3	France	2008	https://clinicaltrials.gov/study/NCT00800995
NCT00706147	SOD	DRUG: Arimoclomol, Placebo	Completed	Amyotrophic Lateral Sclerosis	2 and 3	United States	2016	https://clinicaltrials.gov/study/NCT00706147
NCT00618917	SOD	GENETIC: Manganese Superoxide Dismutase Plasmid Liposome|DRUG: carboplatin, paclitaxel|RADIATION: Radiation Therapy	Terminated	Esophageal / Toxicity	1 and 2	United States	2011	https://clinicaltrials.gov/study/NCT00618917
NCT00264186	SOD	DRUG: LPS intravenous (IV) bolus; rhSOD intraarterially; norepinephrine intraarterially; acetylcholine intraarterially; glyceroltrinitrate (nitroglycerine).	Completed	Inflammation	1	Austria	2005	https://clinicaltrials.gov/study/NCT00264186
NCT06800729	Trx	DRUG: TIX100, an orally available inhibitor of thioredoxin-interacting protein, Placebo	Recruiting	Healthy	1	United States	2025	https://clinicaltrials.gov/study/NCT06800729
NCT06492811	Catalase	DRUG: GAT@F nanoenzyme hydrogel complex	Active, not recruiting	Diabetic Wound	2	China	2025	https://clinicaltrials.gov/study/NCT06492811
NCT06424704	Catalase	DIAGNOSTIC_TEST: Microbiological culture	Not yet recruiting	Otitis Media, Suppurative Chronic / Cholesteatoma / Microbial Colonization	Observational [Patient Registry]	Turkey	2026	https://clinicaltrials.gov/study/NCT06424704
NCT06213857	Catalase, SOD, GPx	DIETARY_SUPPLEMENT: Silymarin|DRUG: Mesalamine|, Azathioprine	Recruiting	Ulcerative Colitis	2	Egypt	2025	https://clinicaltrials.gov/study/NCT06213857
NCT05985278	Catalase	DRUG: [Lu-177]-Catalase	Recruiting	Advanced Malignant Neoplasm	Early Phase 1	China	2026	https://clinicaltrials.gov/study/NCT05985278
NCT05751317	Catalase	DIAGNOSTIC_TEST: culture; staining; biochemical reactions; VITEK; antibiotic sensitivity test; nanoparticles effect; biofilm formation activity; molecular diagnosis.	Unkown status	Evaluate Enterococci by isolating clinical/environmental strains, determining antibiotic susceptibility, assessing biofilm formation, testing how nanoparticles affect antimicrobial resistance and biofilm, and genotyping the isolates to link phenotypes with genetic profiles	Observational	Egypt	2024	https://clinicaltrials.gov/study/NCT05751317
NCT05215444	Catalase, SOD	DIAGNOSTIC_TEST: some prognostic factors	Completed	Aluminum Phosphide Poisoning	Observational	Egypt	2021	https://clinicaltrials.gov/study/NCT05215444

NCT05060120	Catalase, SOD, Trx	DIAGNOSTIC_TEST: biomakers for ovarian torsion|, peritoneal fluid proteins analysis	Unkown status	Ovary Torsion / Adnexal Torsion	Observational	Israel	2024	https://clinicaltrials.gov/study/NCT05060120
NCT04723446	Catalase	DRUG: Corsodyl® Alcohol free -0.2 % Chlorhexidine digluconate|DRUG: Colgate Peroxyl® -1.5% Hydrogen peroxide|OTHER: Oral-B® Gum & Enamel Care -Cetylpyridinium chloride|OTHER: No rinsing	Completed	Covid19 / Coronavirus	Not Applicable	United Kingdom	2021	https://clinicaltrials.gov/study/NCT04723446
NCT04647123	Catalase	OTHER: No intervention was applied to the groups the samples will be collected in terms of the diagnosis (hopeless, periodontitis, gingivitis, healthy)	Unkown status	Periodontitis	Observational [Patient Registry]	Turkey	2021	https://clinicaltrials.gov/study/NCT04647123
NCT04363606	Catalase, SOD, GPx	OTHER: Questionnaires|BIOLOGICAL: blood test|OTHER: Maximal effort test|DEVICE: actigraphy|DEVICE: Neuromuscular evaluation, stool analysis, food diary	Terminated	Chronic Fatigue Syndrome / Intensive Care Unit / Muscle	Not Applicable	France	2022	https://clinicaltrials.gov/study/NCT04363606
NCT04310748	Catalase	DRUG: Total Intravenous Anesthesia(TIVA)|DRUG: Inhalation Anesthesia	Completed	Oxidative Stress / Anesthesia	Not Applicable	Turkey	2021	https://clinicaltrials.gov/study/NCT04310748
NCT04180332	Catalase, SOD	DIAGNOSTIC_TEST: Blood sample	Completed	Periodontal Diseases / Diabetes Mellitus / Enzyme Disorder	Not Applicable	India	2018	https://clinicaltrials.gov/study/NCT04180332
NCT03754010	Catalase	PROCEDURE: Scaling and root planning|DRUG: Hyaluronic acid gel (HA) and SRP, HA mouthrinse and SRP|, HA mouthrinse+gel and SRP	Completed	Periodontitis	1	Turkey	2018	https://clinicaltrials.gov/study/NCT03754010
NCT03621995	Catalase	OTHER: Malondialdehyde and Catalase level measurement	Completed	Oxidative Stress	Observational	Egypt	2018	https://clinicaltrials.gov/study/NCT03621995
NCT03470857	Catalase, SOD, GPx	DIAGNOSTIC_TEST: Patients	Completed	Neoplasms / Antioxidants / Oxidative Stress / Lipid Peroxidation / Inflammation / Lysosome Alteration	Observational	Poland	2021	https://clinicaltrials.gov/study/NCT03470857
NCT02463318	Catalase, SOD	DRUG: Melatonin|OTHER: Hydrogen peroxide	Completed	Multiple Sclerosis / Oxidative Stress	Not Applicable	Iran	2015	https://clinicaltrials.gov/study/NCT02463318
NCT02349685	Catalase	PROCEDURE: H. pylori culture and antimicrobial susceptibility test|DRUG: 14 day PBMT group|DRUG: 14 day MEA group	Completed	Helicobacter Infection	Not Applicable	No location data	2014	https://clinicaltrials.gov/study/NCT02349685
NCT01184339	Catalase	DEVICE: MicroPhage S. aureus/MSSA/MRSA Blood Culture Test	Completed	Bacteremia	Observational	No location data	2010	https://clinicaltrials.gov/study/NCT01184339
NCT00816426	Catalase	DRUG: Rifampicin, Isoniazid, Pyrazinamide, Kanamycin|, Moxifloxacin	Completed	Tuberculosis	1	South Korea	2017	https://clinicaltrials.gov/study/NCT00816426
NCT03364296	Prx	OTHER: Blood Samples	Recruiting	Stroke, Acute	Not Applicable	France	2025	https://clinicaltrials.gov/study/NCT03364296
NCT06859788	GPx	DRUG: Ebselen	Not yet recruiting	Meniere's Disease	3	United States	2026	https://clinicaltrials.gov/study/NCT06859788
NCT06554392	Gpx4	DIAGNOSTIC_TEST: Blood sample	Not yet recruiting	Spondyloarthropathy	Observational [Patient Registry]	No location data	2027	https://clinicaltrials.gov/study/NCT06554392
NCT06394544	GPx	DIETARY_SUPPLEMENT: Brazil Nut	Not yet recruiting	Chronic Kidney Diseases / Chronic Kidney Disease stage3 / Chronic Kidney Disease Stage 3A / Chronic Kidney Disease Stage 3B / Chronic Kidney	Not Applicable	Brazil	2027	https://clinicaltrials.gov/study/NCT06394544

				Disease stage4 / Inflammation / Inflammatory Response / Oxidative Stress / Intestinal Microbiota				
NCT06340633	GPx	DRUG: Ebselen, Placebo	Recruiting	Hearing Loss / Cochlear Trauma	2	United States	2025	https://clinicaltrials.gov/study/NCT06340633
NCT06213857	GPx, SOD	DIETARY_SUPPLEMENT: Silymarin|DRUG: Mesalamine|DRUG: Azathioprine	Recruiting	Ulcerative Colitis	2	Egypt	2025	https://clinicaltrials.gov/study/NCT06213857
NCT05908877	Gpx	OTHER: history and clinical evaluation; nutritional assessment. DIAGNOSTIC_TEST: biological evaluation of the metabolic syndrome and components; ultrasound evaluation of subclinical atherosclerosis and visceral fat storage. BEHAVIORAL: psychological evaluation of the impact of obesity/overweight.	Unkown status	Pediatric Obesity / Metabolic Syndrome / Nonalcoholic Fatty Liver / Atherosclerosis / Psychology / Cardiometabolic Syndrome	Observational	Romania	2024	https://clinicaltrials.gov/study/NCT05908877
NCT05823337	GPx	DIETARY_SUPPLEMENT: selenium+ diet, only diet	Unkown status	Gestational Diabetes / Pregnancy Related	Not Applicable	Turkey	2023	https://clinicaltrials.gov/study/NCT05823337
